# Tregs transduced with a Ro60-reactive TCR have increased immunosuppressive functions in Sjögren’s disease

**DOI:** 10.1016/j.isci.2026.117642

**Published:** 2026-09-28

**Authors:** Zhi Feng Sherman Lim, Rachel M.Y. Cheong, Fabien B. Vincent, Janet Chang, Khai L. Loh, Hanim Halim, Geoffrey Kwai-Wai Kong, Elean S.V. Tay, Cheng Rui Peh, Xiaoyu Jia, Maureen Rischmueller, Yi Tian Ting, Joshua D. Ooi

**Affiliations:** 1 Centre for Inflammatory Diseases, Department of Medicine, School of Clinical Sciences at Monash Health, Monash University, Clayton, VIC, Australia; 2 Monash Macromolecular Crystallisation Platform, Monash University, Clayton, VIC, Australia; 3 Renal Division, Peking University First Hospital, Beijing, China; 4 Institute of Nephrology, Peking University, Beijing, China; 5 College of Health, Adelaide University, Adelaide, South Australia, Australia; 6 Rheumatology Department, The Queen Elizabeth Hospital, Woodville South, SouthAustralia, Australia

**Keywords:** autoimmune disease, Sjögren’s disease, regulatory T cells, cell therapy, translational research

## Abstract

Sjögren’s disease (SjD) is a T cell-driven autoimmune condition strongly associated with the HLA-DRB1∗03:01 allele and autoantibodies against the Ro60 protein. Previous studies have identified deficiencies in regulatory T cells (Treg) in patients, indicating a breakdown in immune tolerance. Current treatments remain limited to symptomatic relief or broad immunosuppression, without addressing the underlying immune dysregulation. In this study, we present the first preclinical evidence supporting a TCR-engineered Treg (TCR-Treg) therapy specifically targeting SjD. We designed a TCR-Treg that recognizes a dominant epitope of the Ro60 antigen (Ro60_369-383_) presented by HLA-DRB1∗03:01 and demonstrate that these cells can selectively suppress autoreactive T cells more effectively than polyclonal Treg. These findings underscore the pathogenic significance of the Ro-HLA-DRB1∗03:01 axis and provide proof-of-concept for a targeted, antigen-specific Treg therapy. This approach offers a promising strategy to restore immune tolerance and represents a potential disease-modifying treatment for genetically defined subsets of patients with SjD.

## Highlights

• Identified a dominant Ro60 epitope restricted by HLA-DRB1∗03:01
• Engineered TCR-Treg targeting the Ro60_369-383_ epitope
• Ro60 Treg show enhanced suppression of autoreactive T cells
• Establishes proof-of-concept for antigen-specific Treg therapy in SjD

## Graphical abstract

**Figure:**
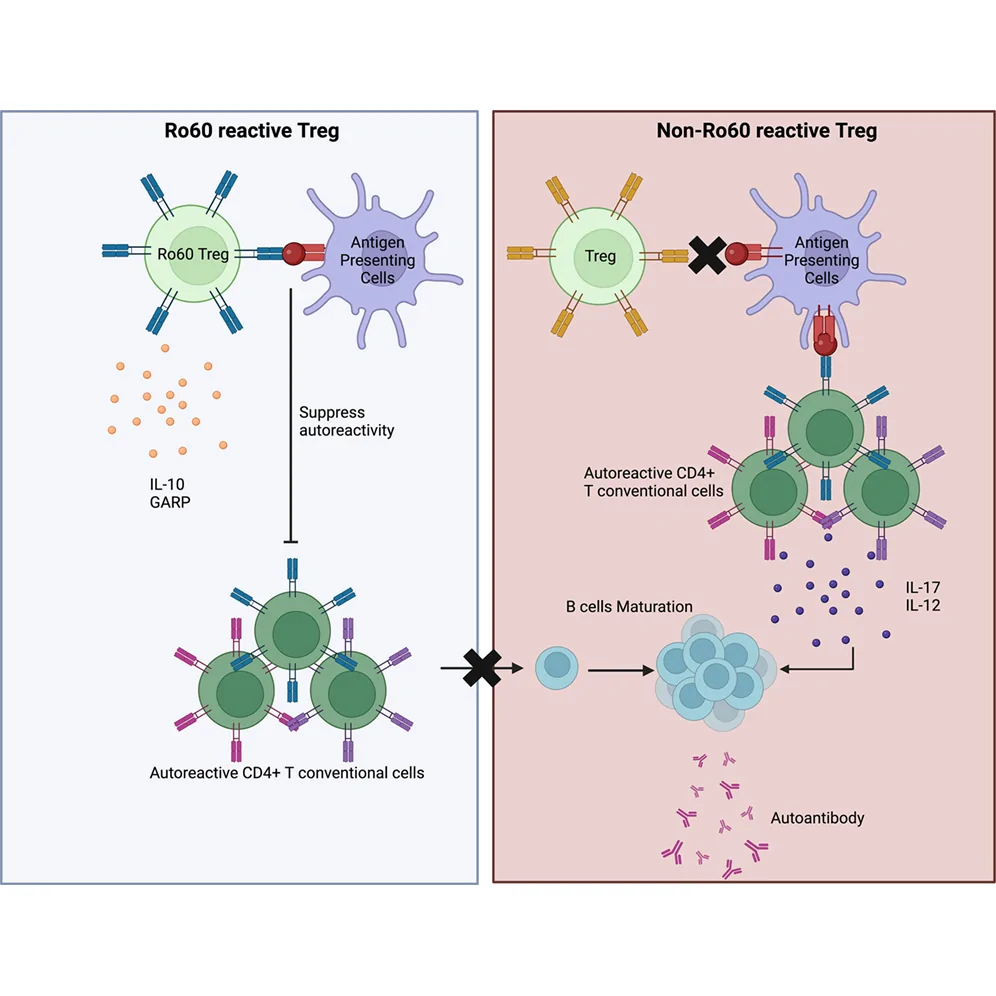

## Teaser

Immunology; Cell biology; Transcriptomics

## Introduction

Sjögren’s disease (SjD) is a systemic autoimmune disease characterized by debilitating glandular inflammation^1^^,^^2^^,^^3^ and a wide spectrum of extra-glandular manifestations that increase morbidity and the risk of B cell lymphoma.^4^ Affecting up to 0.2% of adults,^5^ with a profound female bias (female-to-male ratio of 10:1),^1^^,^^2^^,^^6^ the disease is driven by a complex interplay between genetic predisposition and environmental triggers. The immunopathology hinges on the activation of autoreactive CD4^+^ T cells, which orchestrate lymphocytic infiltration of exocrine glands and promote B cell hyperactivity, leading to the production of hallmark autoantibodies.^2^^,^^7^

A critical factor in SjD pathogenesis is the strong genetic association with the major histocompatibility complex (MHC) class II allele HLA-DRB1∗03:01, which is present in up to 90% of patients of European and Chinese ancestry.^8^^,^^9^^,^^10^ This allele is particularly efficient at presenting peptides derived from the Ro autoantigen, released from apoptotic epithelial cells to pathogenic T cells.^11^^,^^12^ This molecular triad—Ro peptides, HLA-DRB1∗03:01, and autoreactive T cell receptors (TCRs)—represents a central event in the breakdown of immune tolerance and initiation of the autoimmune cascade.

The Ro antigen and its subsequent autoantibodies are not merely markers but active participants in disease pathogenesis.^13^^,^^14^ The Ro antigen itself, comprising Ro60 and Ro52 proteins, can act as the primary immunological trigger when its peptides are presented by antigen-presenting cells.^8^^,^^9^^,^^15^ Preclinical studies have directly confirmed this pathogenic role, demonstrating that the administration of Ro peptides can induce autoimmune-like responses in the salivary glands of animal models.^16^ The resulting anti-Ro autoantibodies, found in approximately 70% of patients, are strongly associated with disease progression and severity.^17^^,^^18^ In particular, the presence of anti-Ro antibodies is linked to more severe systemic phenotypes, extra-glandular involvement, and an increased risk of complications, highlighting their contribution to the progression of SjD.^19^

Despite this understanding, the current therapeutic landscape for SjD is inadequate. Treatment is largely limited to symptomatic relief-like tear and salivary substitutes, and off-label use of broad-spectrum immunosuppressants, neither of which halts disease progression or addresses the underlying cause.^20^ Although emerging therapies targeting B cells or T cell co-stimulation—such as rituximab, remibrutinib, and frexalimab—show considerable promise, a substantial unmet need remains for treatments capable of restoring self-tolerance in a targeted and specific manner.^21^^,^^22^^,^^23^^,^^24^^,^^25^ Regulatory T cells (Treg) are essential for maintaining peripheral tolerance, but their function and number are known to be impaired in SjD patients,^26^ contributing to unchecked autoimmunity.^27^^,^^28^^,^^29^

Antigen-specific engineered Treg, shown in other autoimmune diseases that share immunopathological pathways such as systemic lupus erythematosus and type 1 diabetes to possess superior suppressive capacity over polyclonal Treg, represent a promising therapeutic avenue.^30^^,^^31^^,^^32^ Ro60, encoded by TROVE2, is a major autoantigen in SjD and acts as a checkpoint for misfolded RNA through its ribonucleoprotein complex with Y RNAs.^21^ Its peptides, particularly Ro60_369–383_ and Ro60_225–239_, could potentially be presented by HLA-DRB1∗03:01 and are recognized by autoreactive T cells,^33^ yet no structural data currently exist for Ro60 peptide presentation by this allele beyond the invariant CLIP peptide structure.^34^ This knowledge gap underscores the need for structural and functional studies to guide therapeutic design.

We hypothesized that restoring antigen-specific regulation at the origin of the autoimmune response could be a powerful therapeutic strategy. Here, we report the development and preclinical *in vitro* validation of a novel TCR-engineered Treg (Ro-Treg). This therapy is specifically designed to recognize a key pathogenic epitope of the Ro antigen, herein designated Ro60_369-383_, when presented by the high-risk HLA-DRB1∗03:01 allele. We aimed to demonstrate that this targeted approach can selectively suppress the autoimmune response, offering a potential disease-modifying therapy for SjD.

## Results

### Ro60_369-383_ showed greater immunogenicity than Ro60_225–239_

To determine the immunogenicity of candidate peptides previously reported by Szymula et al., Ro60_225–239_ and Ro60_369–383_ were tested in Tconv co-culture assays using two different healthy donors. Ro60_369–383_ elicited the highest level of T cell proliferation compared with Ro60_225–239_, exceeding baseline proliferation observed in the absence of peptide stimulation (Figure 1). Based on these results, Ro60_369–383_ was selected as the epitope for further analyses.

**Figure 1:**
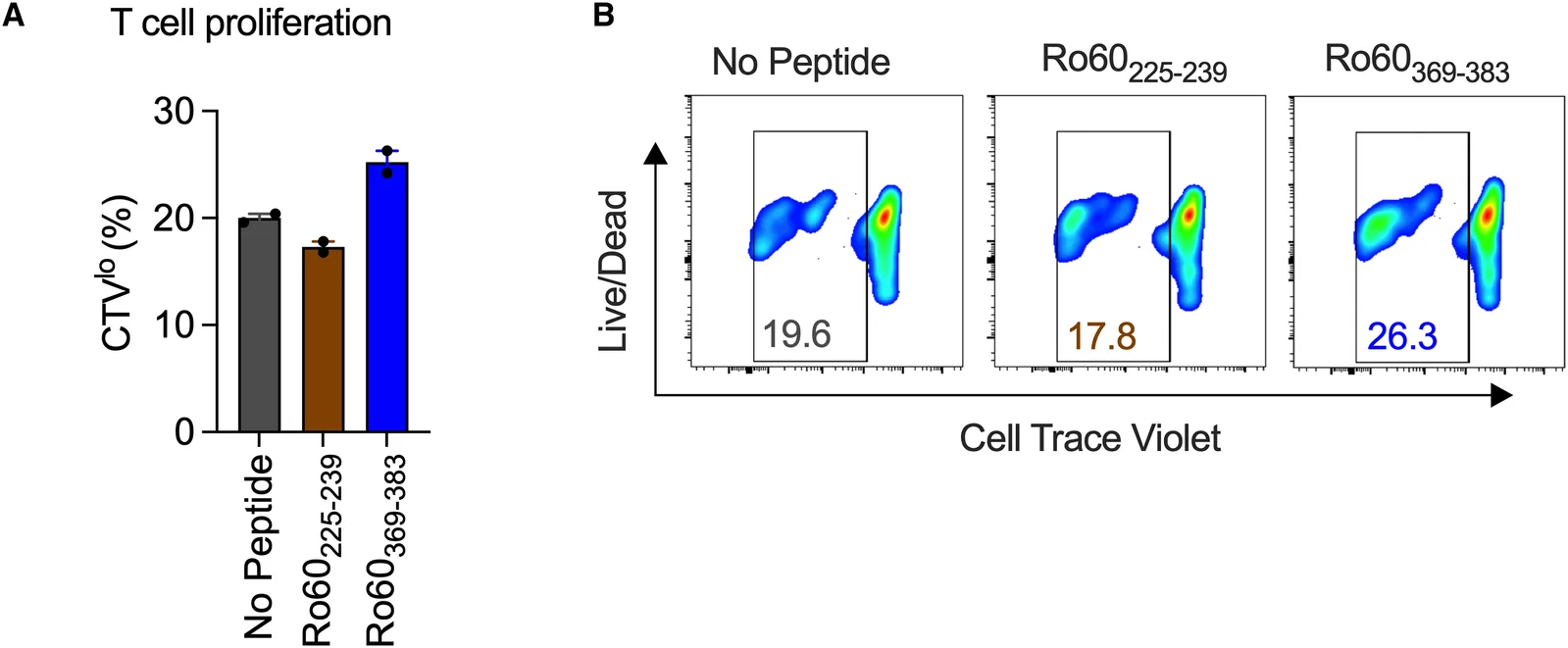
Ro60_369–383_ exhibits higher immunogenicity (A Quantification of T cell proliferation, shown as percent CTV dilution, comparing responses to Ro60_225–239_, Ro60_369–383_, and the no-peptide control. (B) Representative flow cytometry plots from the co-culture assay showing T cell proliferation following exposure to no peptide, Ro60_225–239_, or Ro60_369–383_. Dot points represent biological replicates.

#### Crystal structure of Ro60_369-383_ in complex with HLA-DR3

To enable structural studies of Ro60_369–383_ binding to HLA-DR3, we attempted recombinant expression of the peptide-MHC complex in mammalian cells. However, yields of HLA-DR3– Ro60_369–383_ averaged only ∼0.3 mg/L, insufficient for crystallization (Figures 2 and S1). Recombinant expression of HLA-DR3 has long posed technical challenges due to its low yield.^34^ To overcome this, we expressed HLA-DR3 in complex with the invariant CLIP peptide and introduced stabilizing point mutations based on the predicted HLA-DR3 binding-pocket pref.^35^ Substituting aspartic acid at Pocket 4 from alanine (P4D) or lysine at Pocket 6 from proline (P6K) markedly improved yields, with CLIP_P6K reaching ∼1.3 mg/L and CLIP_P4D achieving ∼11 mg/L (Figure 2). The CLIP_P4D complex exhibited a greater than 10-fold increase in HLA-DR3 protein production was chosen for downstream work and successfully exchanged with Ro60_369–383_. The backreflection curves confirmed that the P4D substitution greatly stabilized the complex as the increase in backreflection commonly attributable to protein aggregation or precipitation was abolished (Figure 2). These findings demonstrate that rationally designed point mutations can enhance the stability and expression of soluble HLA-DR3, enabling structural characterization of low-yield peptide-MHC complexes.

**Figure 2:**
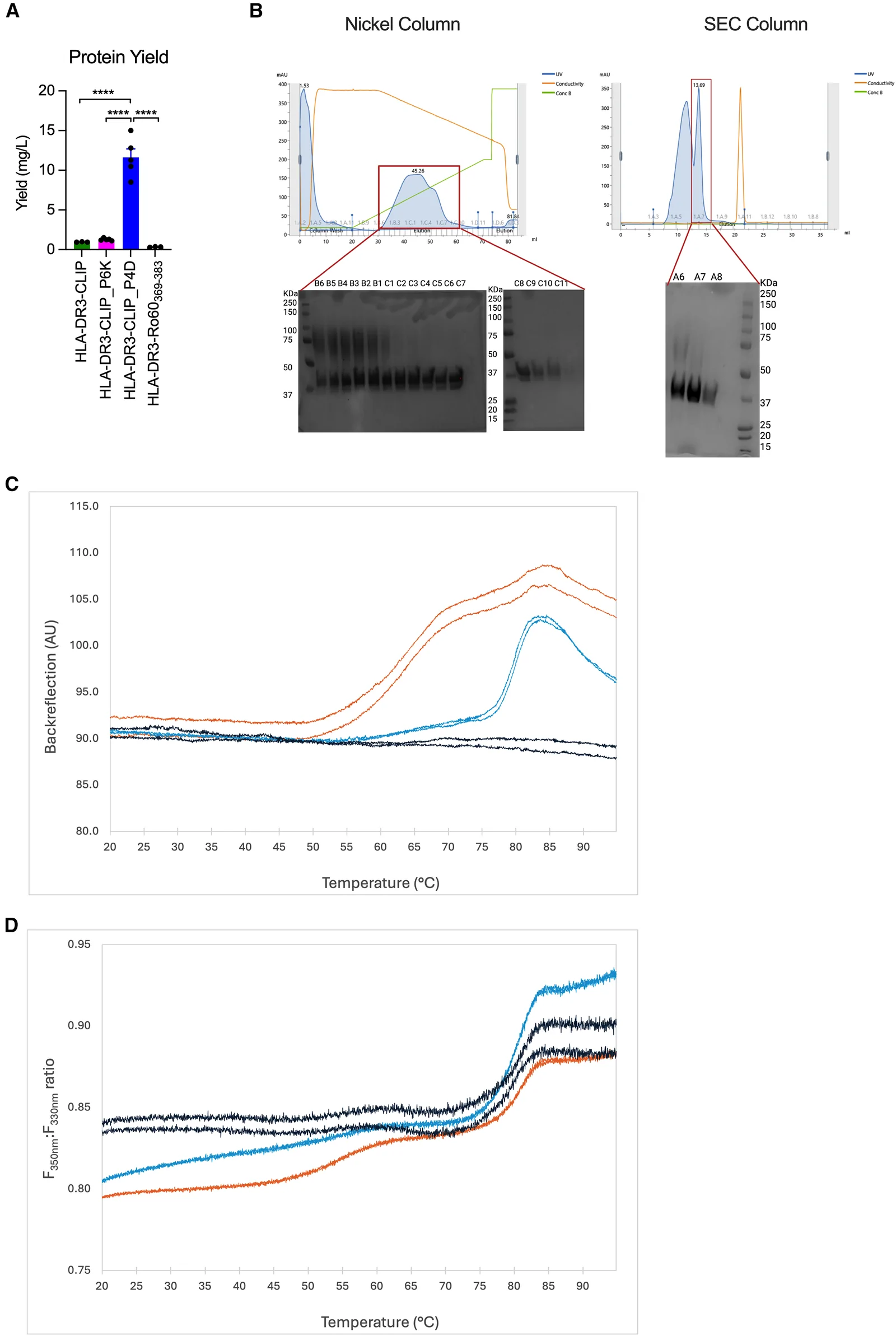
HLA-DR3-CLIPP4D complex demonstrates the highest protein yield and increased thermal stability (A) Bar graph summarizing protein expression levels of four different HLA-DR3 complexes expressed in a mammalian system. (B) Representative purification profile of HLA-DR3-CLIPP4D using nickel purification column and SEC purification column to remove undesired protein contaminants. (C) Comparison of the backreflection curves of HLA-DR3-CLIP (orange), HLA-DR3-CLIP_P6K (light blue), and HLA-DR3-CLIP_P4D (navy blue) in duplicate. The relatively flat curves for HLA-DR3-CLIP_P4D indicate its enhanced stability. (D) Comparison of the thermal melting of HLA-DR3-CLIP (orange), HLA-DR3-CLIP_P6K (light blue), and HLA-DR3-CLIP_P4D (navy blue) in duplicate as determined by the ratio of exposed and buried aromatic residues measured by fluorescence emission at 350 and 330 nm, respectively. The relatively flat section from 20 °C to 75 °C for HLA-DR3-CLIP_P4D is another indication of its greater stability. ∗∗∗∗*p* < 0.0001 by ANOVA.

Using this optimized platform, we determined the crystal structure of HLA-DR3 bound to the immunodominant Ro60_369–383_ peptide (PDB 9YZ3) at 2.87 Å resolution (R_work 23.7%, R_free 29.9%). In the resulting structure, the Ro60_369–383_ peptide adopts a canonical register, with the 9-mer binding core defined as FLLAVDVSA. Three residues: P2-Leucine, P5-Valine, and P8-Serine were solvent-exposed and positioned for TCR recognition (Figure 3). This structure not only validates Ro60_369–383_ as the dominant T cell epitope in HLA-DR3 but also reveals candidate residues likely to engage autoreactive TCRs in SjD.

**Figure 3:**
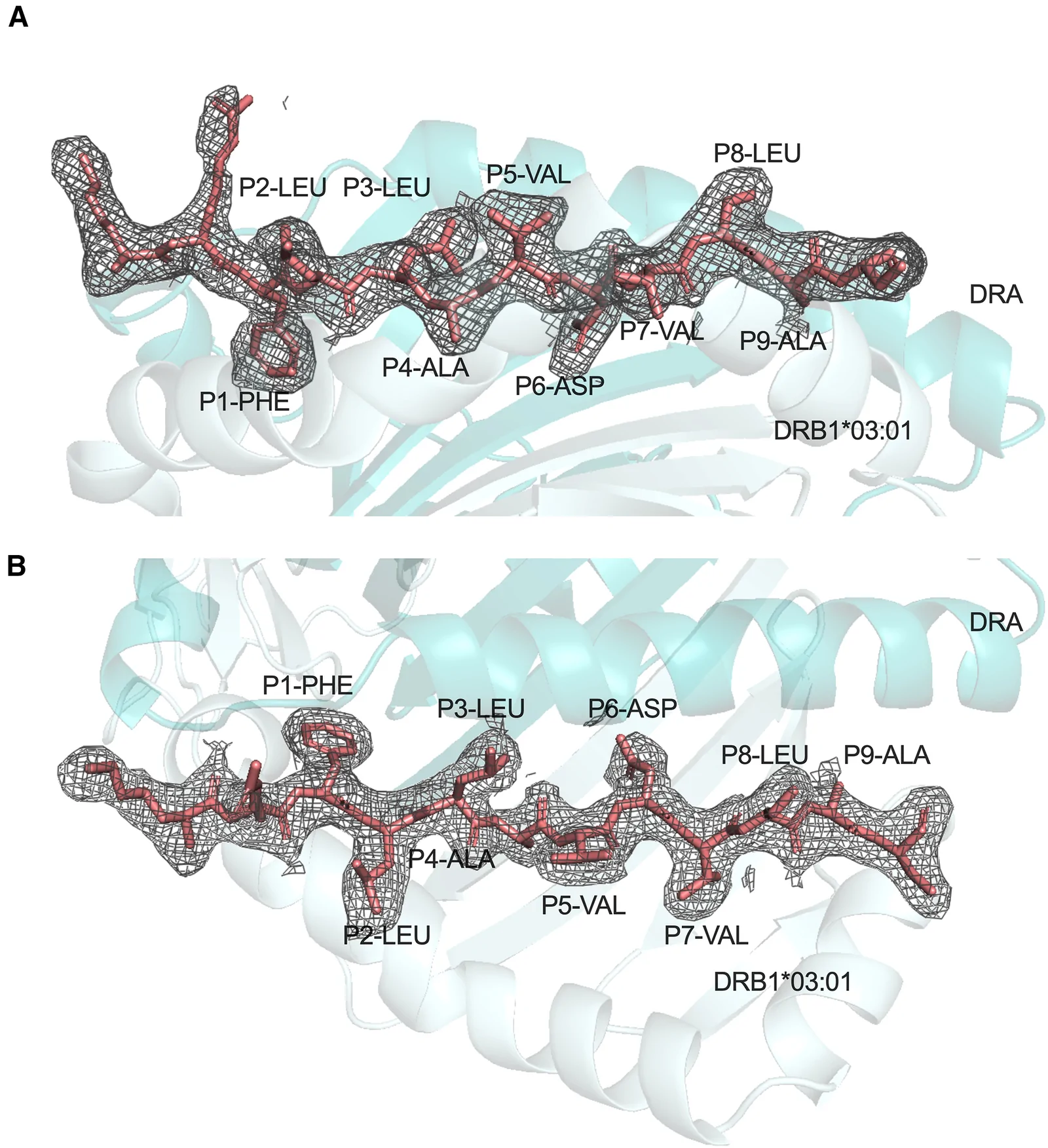
Crystal structure of HLA-DRB1∗03:01 binary crystal structure in complex with Ro60_369-383_ (PDB 9YZ3) (A) Side view of Ro60_(369–383)_ bounded on HLA-DRB1∗03:01 across the binding groove with P2-LEU, P5-VAL and P8-SER accessible for TCR binding. (B) Top view of HLA-DRB1∗03:01 bounded with Ro60_(369–383)_ in a 2Fo-Fc electron density map, contoured at 1σ.

#### Identification of a Ro60_369-383_-specific Treg-derived TCR

We identified Ro60_369–383_ as the immunodominant T cell epitope presented by HLA-DR3 and uncovered a corresponding TCR derived from thymically originated Treg. Using an *in vitro* co-culture assay of CD4^+^ T cells and autologous monocyte-derived dendritic cells from a healthy HLA-DR3 donor, followed by single-cell RNA sequencing, we detected a dominant clonally expanded TCR, designated TCR1, which had the highest clonal frequency among all recovered sequences (Figure 4). Transcriptomic profiling revealed that TCR1^+^ clones expressed hallmark genes of thymically derived Treg (tTreg), including elevated Forkhead Box P3 (FOXP3), the defining transcription factor of Treg,^36^^,^^37^ and IKZF2 (Helios), which is essential for Treg development and function,^38^^,^^39^ compared with other T cell populations (Figure 4). Collectively, these data strongly support that TCR1 originates from a regulatory T cell lineage.

**Figure 4:**
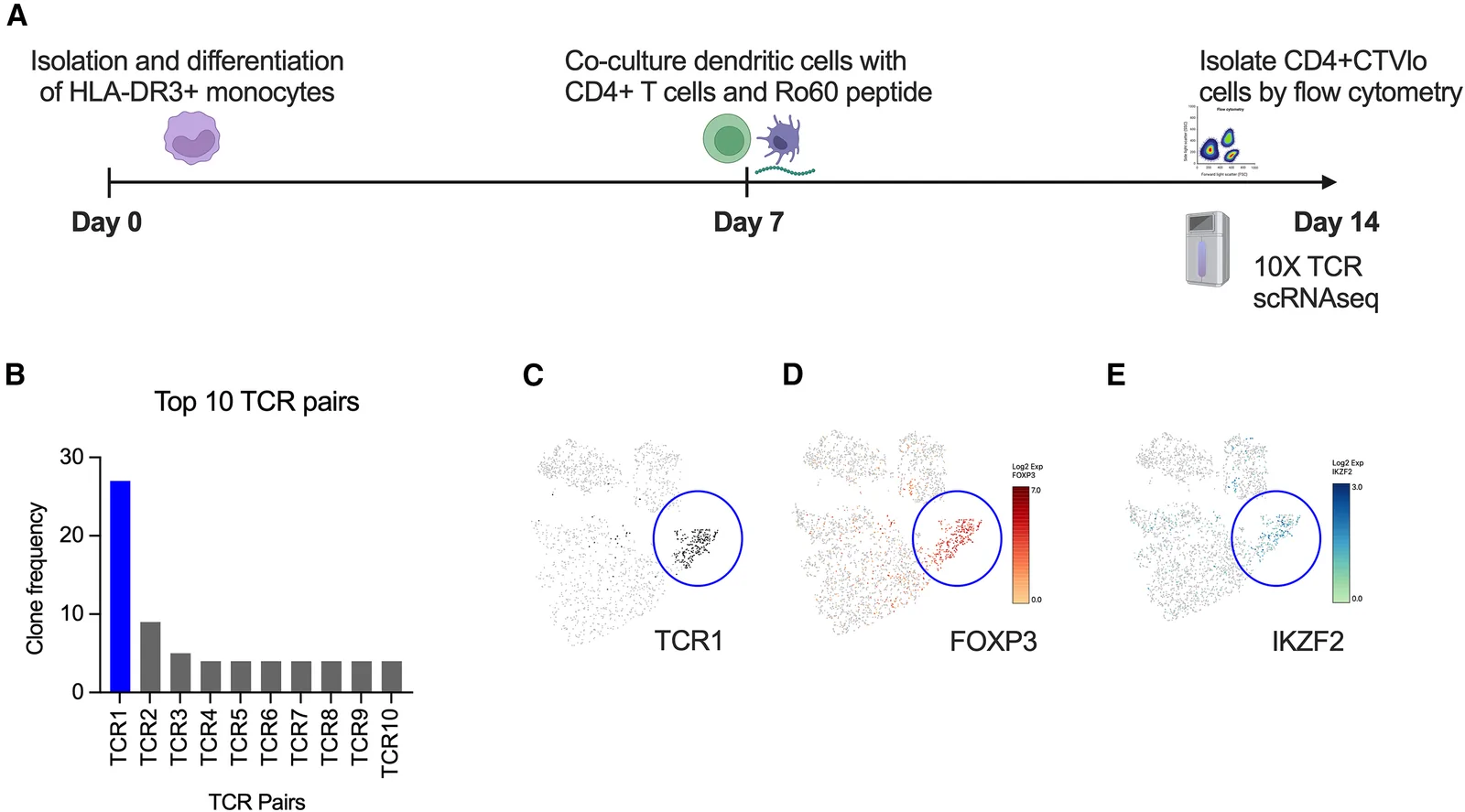
Identification of a Treg-derived TCR specific to Ro60_369-383_ through single-cell RNA sequencing in HLA-DR3^+^ healthy control (A) Experimental timeline of the co-culture assay used to identify the TCR. (B) Depicting top 10 TCRs discovered along with their clonal frequencies from 10X TCR single-cell sequencing data. (C) t-SNE plot showing CD4^+^ T cells expressing TCR1 (black). (D) t-SNE plot of FOXP3 expression, confirming that the CD4^+^ T cells are regulatory T cells (red). (E) t-SNE plot of IKZF2 expression, also known as Helios, is a transcription factor that plays a vital role in development of thymic-derived Treg (blue).

#### Optimization of TCR1 transduction and vector copy number

Safe and efficient TCR transduction must comply with US Food and Drug Administration (FDA) guidelines, which limit the viral vector copy number (VCN) to fewer than five copies per cell to minimize the risk of insertional mutagenesis.^40^ Determining an appropriate multiplicity of infection (MOI) is therefore critical to balancing transduction efficiency with safety. We evaluated a range of MOIs, specifically 1, 2, 3, 5, and 8, and observed that increasing the MOI led to higher GFP positivity (21%–31%), indicating improved transduction efficiency. However, TCR1 surface expression remained stable at 5–9% across all MOIs tested (Figures 5 and S2). qPCR-based VCN analysis confirmed that only MOIs of 1 and 2 achieved VCN values ≤5, consistent with regulatory standards. Based on these findings, MOI 2 was selected for subsequent experiments as it provided an optimal balance of safety, transduction efficiency, and stable TCR1 surface expression (Figure 5).

**Figure 5:**
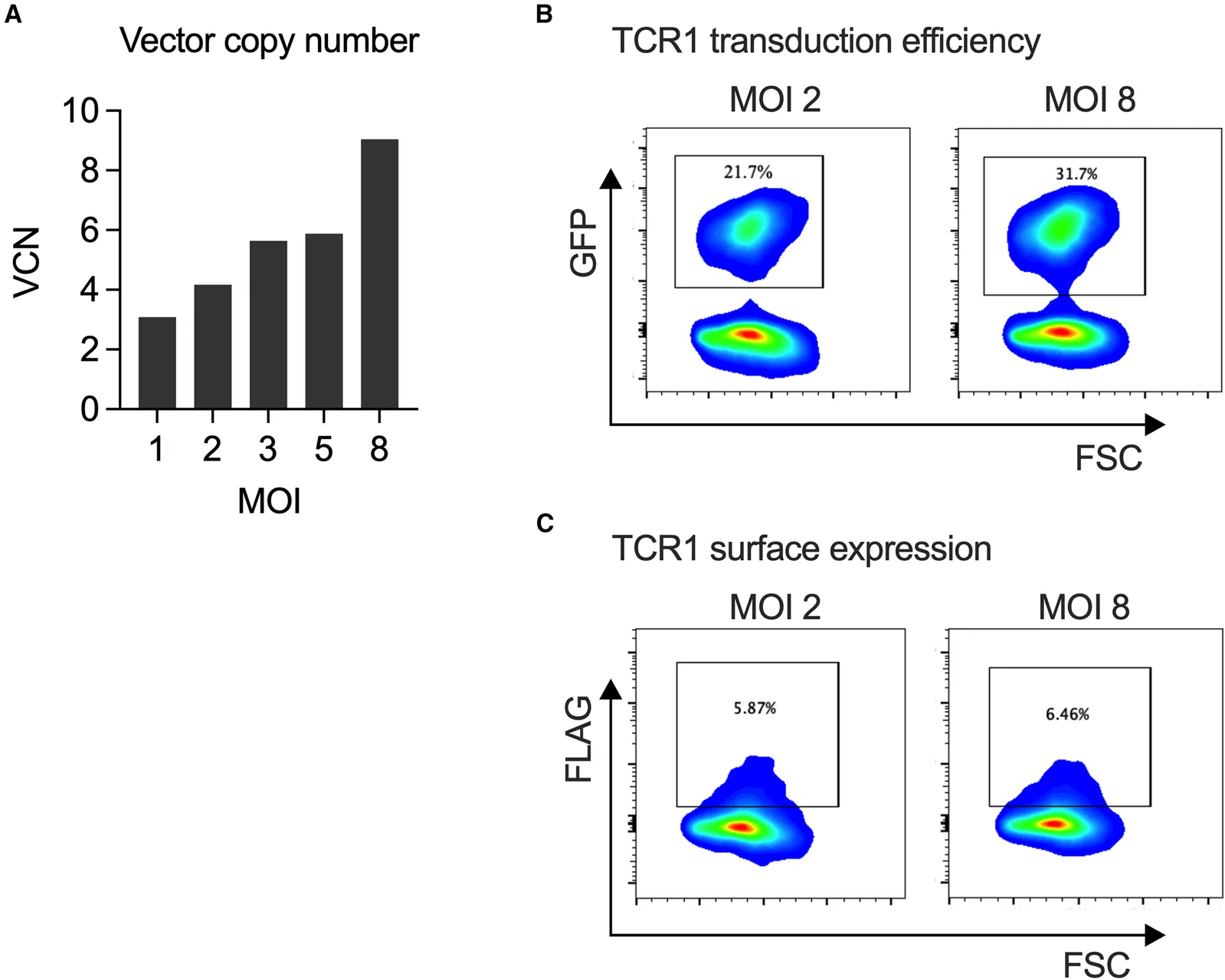
Lentiviral transduction of TCR1 into human Treg using an MOI of 2 is sufficient to yield high transduction efficiency, and good surface expression while keeping the VCN below 5 (A) Bar graph displaying the VCN corresponding to MOIs of 1, 2, 3, 5, and 8. (B and C) Flow cytometry plots showing GFP% and FLAG% for MOI 2 and MOI 8, demonstrating consistent transduction efficiency and TCR1 expression across these MOIs.

### Comparable TCR1 transduction efficiency in healthy donors and SjD patients

We next compared TCR1 transduction between Treg from healthy donors (HCs, *n* = 4) and SjD patients (*n* = 5). In HCs, transduction efficiencies ranged from 23% to 27% GFP^+^ with consistent 5%–7% TCR1 surface expression (Figures 6 and S4–S5). SjD patient Treg exhibited broader variability (3%–20% GFP^+^; 6%–18% surface expression of TCR1), but no statistically significant differences were observed between groups. These results demonstrate that TCR1 can be successfully engineered into both HC and SjD patient Treg (Figure 6).

**Figure 6:**
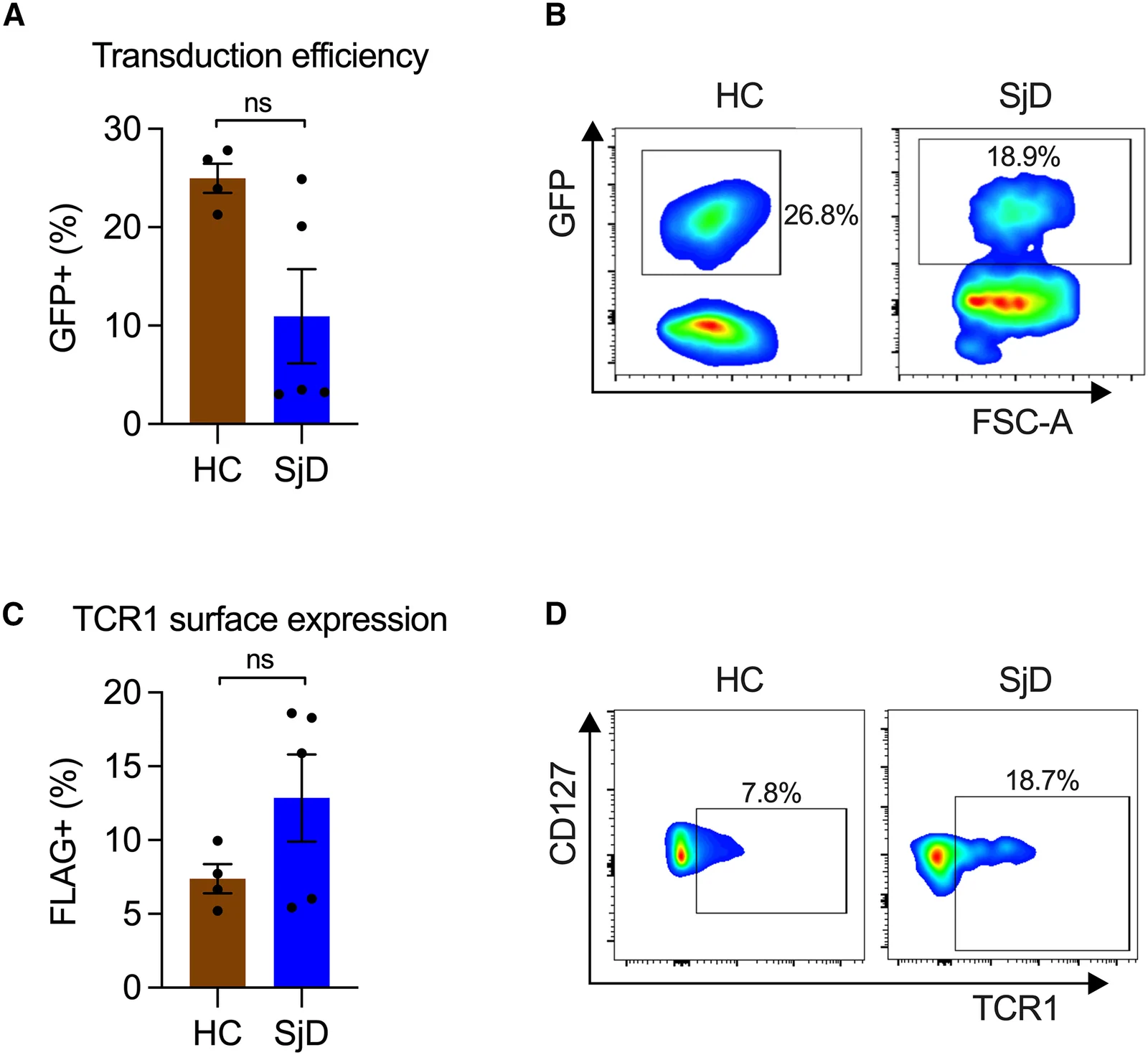
TCR1 is expressible and transducible in both Treg from healthy controls (HC) and SjD patients (A) Statistical comparison of transduction efficiency between SjD patients and HC. (B) Representative flow cytometry plots depicting transduction efficiency of Treg from SjD and healthy control. (C) Statistical comparison of TCR1 expression between SjD patients and healthy controls. (D) Representative flow cytometry plots of TCR1 surface expression in Treg from SjD patient and healthy control. ns, *p* > 0.05 by ANOVA.

### SjD patients’ Treg engineered with TCR1 (Ro60-Treg) showed enhanced activation suppression function

We next evaluated the activation, proliferation, and suppressive function of Ro60-reactive Treg (Ro60-Treg) from SjD patients (*n* = 5). When stimulated with the Ro60_369–383_ peptide, Ro60-Treg from five patients proliferated significantly more than their non-engineered counterparts (mock-Treg), as measured by CTFR dye dilution (Figures 7 and S7). Ro60-Treg also showed higher expression of activation markers, including CD69 (an early T cell activation marker) and glycoprotein A repetitive pattern (GARP, a membrane protein associated with Treg activation) (Figure 7). Together, these results indicate that Ro60-Treg display heightened activation and a corresponding increase in proliferative capacity *in vitro*.

**Figure 7:**
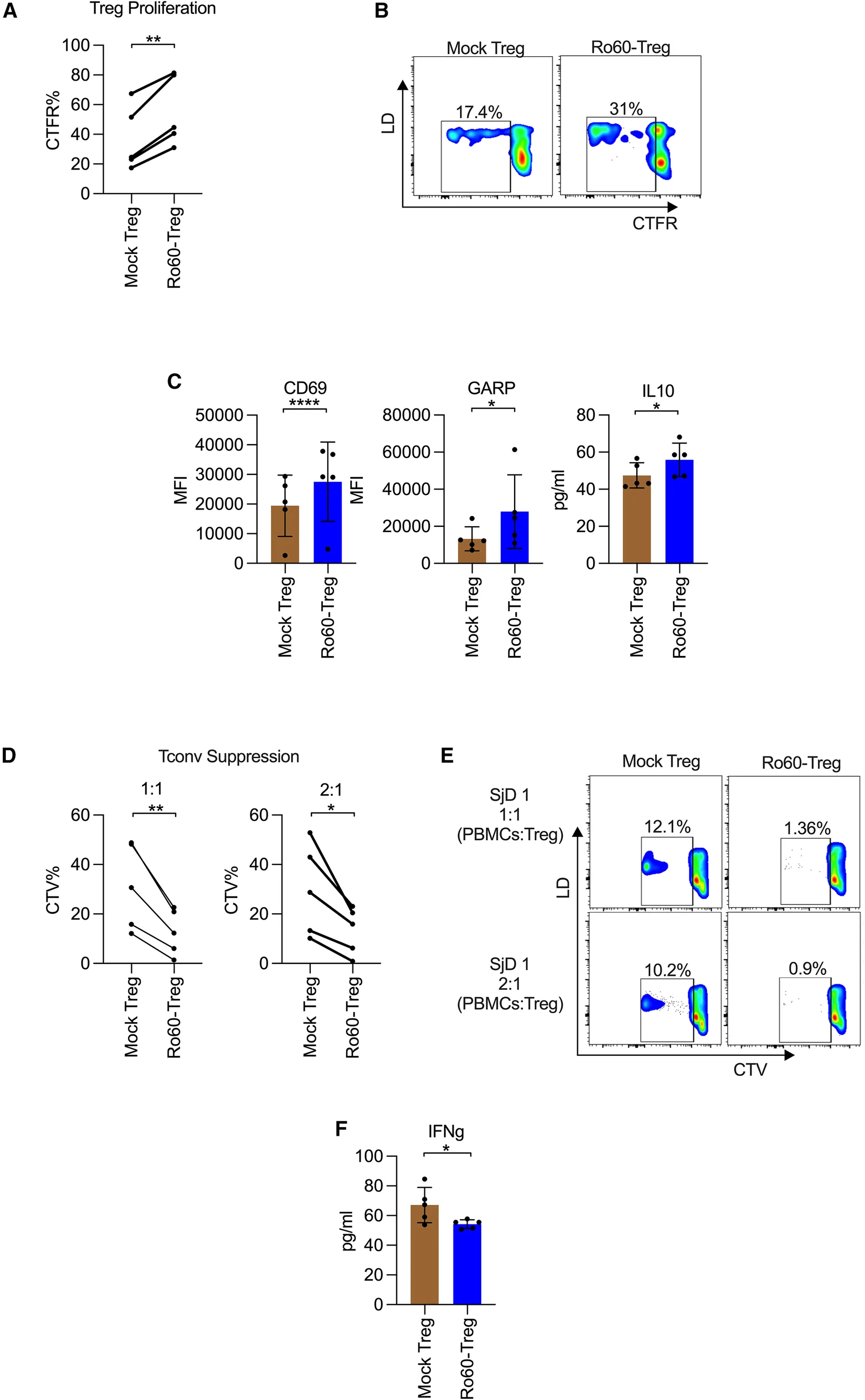
Ro60-Treg exhibit enhanced activation and suppressive function (A) Statistical comparison of proliferation between engineered and non-engineered Treg across all five SjD patients. (B) Flow cytometry plots showing activation levels, as indicated by CTFR%, in SjD 2. (C) Bar graph showing statistical analysis of CD69, GARP, and IL10. (D) Statistical comparison of CTV% between engineered and non-engineered Treg across all five SjD patients at ratio 1:1 and 2:1 (Tconv to Treg). (E) Flow cytometry plots of SjD 1 at two different ratios of 1:1 and 2:1 (Tconv to Treg), demonstrating suppressive function as indicated by CTV%. (F) Bar graph showing statistical analysis of IFNg in the suppression assay. *∗p <* 0.05, *∗∗p <* 0.01, and *∗∗∗∗p <* 0.0001 by ANOVA.

In suppression assays using Tconv cells, peripheral blood mononuclear cells (PBMCs) or CD25^−^ cells as responder populations, Ro60-Treg consistently demonstrated significant greater suppressive capacity across all patient samples (Figures 7, S8, and S9). These findings also show that CD25^−^ cells and PBMCs can serve as alternative responder populations for suppression assays, with measurable outcome (Figures 7, S8, and S9). This effect remained significant when data were pooled. Cytokine profiling further revealed increased IL-10 and reduced IFN-γ in co-cultures with Ro60-Treg, confirming an enhanced immunoregulatory phenotype (Figure 7).

In summary, we identified Ro60_369–383_ as the immunodominant T cell epitope in the context of HLA-DR3 and discovered a corresponding TCR (TCR1) derived from thymically-originated Treg cells using single-cell RNA sequencing. Despite challenges in producing soluble HLA-DR3-Ro60 complexes, engineering HLA-DR3-CLIP with stabilizing mutations enabled efficient peptide exchange, yielding sufficient protein for structural validation. Notably, this is the first structure of HLA-DR3 bound to an autoantigenic peptide, as previous structural data have been limited to complexes with CLIP peptides. This advancement provides a valuable foundation for epitope characterization and the design of antigen-specific TCR therapies. TCR1 was successfully engineered into Treg from both healthy donors and SjD patients with consistent transduction efficiency, stable surface expression, and within an FDA-compliant VCN range. Importantly, Ro60-Treg exhibited approximately 2-fold enhanced suppression of Ro60-driven inflammatory responses compared with non-engineered Treg, demonstrating robust antigen-specific regulatory function across both donor types. This enhanced functionality is likely attributable to the antigen-specific engagement of the introduced TCR1, highlighting the therapeutic potential of engineered Ro60-Treg as a precision Treg-based strategy for SjD.

## Discussion

SjD is a systemic autoimmune condition marked by debilitating glandular inflammation and a wide range of extra-glandular manifestations. Approximately 70% of patients are anti-Ro seropositive, and up to 90% carry the HLA-DR3 allele; notably, 81.8% present with systemic involvement at diagnosis.^1^^,^^8^^,^^17^^,^^41^^,^^42^ Our engineered TCR-Treg directed against Ro60_369-383_ exhibited robust, antigen-specific suppressive activity *in vitro*, effectively inhibiting pathogenic T cell responses. These results set the stage for a precision cell therapy aimed at restoring immune tolerance in SjD.

This work validates the Ro/HLA-DRB1∗03:01 axis as a critical driver of SjD pathogenesis and as a high-value therapeutic target. Importantly, we present the first structural characterization of HLA-DR3 bound to an autoantigenic peptide, as previous data were limited to only the CLIP peptide complexes. This advance provides a crucial foundation for epitope mapping and for designing antigen-specific TCR therapies.

Unlike conventional systemic immunosuppressants or biologics that broadly dampen immunity, our TCR-Treg are designed to act only where their peptide-MHC target is present, primarily in inflamed tissues presenting autoantigens.^43^^,^^44^ This specificity offers the promise of improved efficacy at disease sites while minimizing the risks of systemic immunosuppression, an especially important safety consideration in a patient population already predisposed to lymphoma.^45^^,^^46^^,^^47^ Furthermore, this strategy aims to promote a durable immune reset rather than transient suppression.

The underlying immune dysregulation in SjD involves the formation of tertiary lymphoid structures within salivary glands, where stromal and epithelial cells, along with follicular helper T (Tfh) cells, orchestrate local immune activation.^48^ Th1-derived IFN-γ promotes BAFF production by antigen-presenting cells, enhancing B cell survival and facilitating the persistence of autoreactive clones.^49^ Simultaneously, IL-21 from Tfh cells promotes B cell differentiation and sustained autoantibody production. These features highlight the pivotal role of autoreactive T cells in both initiating and perpetuating SjD pathogenesis.^50^

This work addresses a critical unmet need for disease-modifying therapies in SjD. The limited efficacy of polyclonal Treg therapies in other autoimmune diseases underscores the importance of antigen specificity to direct immunosuppressive activity more precisely.^51^^,^^52^^,^^53^ Our TCR-Treg approach is particularly well-suited to SjD, as it targets the initiating T cell response to an intracellular autoantigen presented by MHC class II—positioning the intervention upstream of B cell activation and autoantibody production.^1^^,^^2^^,^^54^^,^^55^ This strategy offers a more fundamental correction of immune dysregulation than downstream approaches such as B cell depletion or cytokine blockade.

Clinical translation of this therapeutic approach will require addressing several challenges. SjD is characterized by heterogeneity in autoantigen and epitope recognition, which may result in variable patient responses; thus, initial clinical studies should focus on HLA-DRB1∗03:01-positive individuals with confirmed T cell reactivity to the Ro60_369–383_ epitope. To support this strategy, we engineered a stabilized HLA-DR3_CLIP construct that enables efficient peptide exchange and production of sufficient protein for structural validation, while also facilitating downstream applications such as tetramer or dextramer development to identify and track autoreactive T cells. This approach may also extend to systemic lupus erythematosus, where HLA-DRB1∗03:01 is prevalent,^30^ although further validation is required to determine its capacity to suppress inflammatory responses in this setting.

We established manufacturing-relevant quality control measures with a particular emphasis on VCN to meet regulatory requirements and support future clinical translation. Quantification of VCN, together with transgene expression profiling in engineered Treg, confirmed stable genomic integration with copy numbers maintained within accepted safety thresholds and demonstrated consistent, physiologically appropriate expression of the introduced TCR. Collectively, these data highlight the reproducibility, potency, and safety of the product, providing a strong foundation for regulatory compliance and advancement toward clinical application.

Ensuring the long-term phenotypic and functional stability of engineered Treg both *in vitro* and *in vivo* will be critical, particularly to mitigate the risk of conversion into pro-inflammatory effector cells. Future studies will therefore focus on systematically evaluating the phenotype and stability of the engineered Treg across these settings. Notably, prior work from our group, including Eggenhuizen et al., has already demonstrated that engineered Treg can retain suppressive function and phenotypic stability over extended periods, providing a strong foundation and rationale for the continued development of this approach in chronic autoimmune diseases.^30^^,^^56^^,^^57^ Note also that in Eggenhuizen et al., the engineered TCR was also obtained from healthy individuals.

Nonetheless, we observed variability in transduction efficiency and transgene expression between Treg derived from SjD patients and healthy controls. This aligns with previous findings in autoimmune diseases such as SLE and SjD, where Treg exhibit impaired functionality and reduced cellular fitness.^7^^,^^26^ Elevated levels of reactive oxygen species (ROS) and oxidative stress have been observed in autoimmune Treg have been implicated in mitochondrial and metabolic dysfunction, further impairing viral transduction and stable transgene integration.^58^^,^^59^^,^^60^ Hence, alternative non-viral gene editing strategies—such as CRISPR/Cas9-mediated targeted integration—may offer a promising solution. Emerging evidence suggests that CRISPR-based approaches can enhance transgene expression and genomic stability in T cells,^61^^,^^62^ potentially overcoming the limitations posed by traditional viral vectors.

TCR repertoires are highly individualized due to differences in HLA alleles, V(D)J recombination, and antigen exposure.^63^ Therefore, the transgenic TCR identified in this study may not necessarily represent a dominant or shared clonotype in other individuals. scRNA sequencing identified this TCR as the most clonally expanded Treg-derived clonotype in the donor analyzed, comprising 27 clones, supporting its biological relevance within this donor. Nevertheless, whether similar Ro60-specific Treg clonotypes are shared across individuals with SjD and healthy controls remains to be determined.

The identification of recurrent TCR sequences across donors would support the presence of a public TCR response. However, the absence of identical clonotypes would not necessarily preclude broader applicability, as the same epitope may be recognized by distinct TCR sequences through convergent antigen recognition. Future studies involving larger and more diverse donor cohorts will therefore be important to determine the extent of inter-individual variability in Ro60-reactive TCR repertoires and whether similar antigen-specific Treg clonotypes can be reproducibly identified across individuals. Such studies will also help determine whether the selected TCR consistently exhibits superior suppressive activity across donors with SjD. Validation in relevant *in vivo* models of SjD will subsequently be required to further assess its suppressive capacity and potential phenotypic stability.

In conclusion, our development of a Ro60_369-383_-specific TCR-Treg represents a major step forward to a targeted, tolerance-inducing therapy for SjD patients who are HLA-DRB1∗03:01 positive. By acting at the root of the autoimmune response, this strategy has the potential to transform treatment for SjD. Ro60-Treg may represent a viable strategy to re-establish immune tolerance and prevent disease progression, while also serving as a model for extending antigen-specific Treg therapies to other HLA-linked autoimmune conditions.

### Limitations of the study

While this study strongly demonstrates that antigen-specific Treg exhibit enhanced suppressive capacity compared with their polyclonal counterparts, thereby advancing therapeutic strategies for SjD, several aspects warrant further investigation. The availability of patient-derived samples, while sufficient to support the current findings, could be expanded in future studies to strengthen statistical power and enable more comprehensive comparisons. In addition, although the *in vitro* assays provide strong evidence of therapeutic potential, extending these findings into *in vivo* models will be an important next step to further validate functional efficacy within a more physiologically relevant context. Collectively, these future directions will help to reinforce and extend the translational potential of this approach in SjD.

## Resource availability

### Lead contact

Further information and requests regarding resources and analyses should be directed to and will be fulfilled by the lead contact, Joshua D. Ooi (joshua.ooi@monash.edu).

### Materials availability

Due to ethical regulations and institutional policies, the availability of human samples and related materials generated in this study may be subject to restrictions. Requests for materials should be directed to the lead contact and reviewed by the Ethics Committee of Monash University and Queen Elizabeth Hospital.

### Data and code availability

• Protein crystal structure data have been deposited at the PDB (https://doi.org/10.2210/pdb9yz3/pdb) and 10X scRNA-seq data deposited at the NCBI Sequence Read Archive (SRR40145114), Accession number: PRJNA1512058.
• This paper does not report original code.
• Any additional information required to reanalyze the data reported in this paper is available from the lead contact upon request.

## Acknowledgments

The authors wish to acknowledge the traditional owners of the lands on which they live and work and pay respects to their Elders past, present, and emerging. This work was supported by the 10.13039/501100000925NHMRC, Australia, and the 10.13039/100025603Long Foundation. 10.13039/501100015418F.B.V. is supported by funding from 10.13039/501100000925National Health and Medical Research Council (Emerging Leadership 1, grant 1196112) and has received support from the Rebecca L. Cooper 10.13039/501100009187Medical Research Foundation and 10.13039/501100000940Arthritis Australia. 10.13039/501100015418F.B.V. has received support from 10.13039/100015756Janssen-Cilag, 10.13039/100004325AstraZeneca and CSL for projects, and from 10.13039/100004319Pfizer, 10.13039/100012051Lupus Research Alliance, and SomaLogic for conference or meeting sponsorship.

## Author contributions

Z.F.S.L., conceptualization, data curation, formal analysis, investigation, methodology, project administration, validation, visualization, writing – original draft, writing – review and editing, resources, and software; R.M.Y.C., investigation, methodology, and writing – review and editing; F.B.V., data curation and writing – review and editing; J.C., investigation and resources; K.L.L., investigation, methodology, and writing – review and editing; H.H., investigation; G.K.-W.K., investigation; E.S.V.T., investigation; C.R.P., investigation; X.J., investigation; M.R., resources and writing – review and editing; Y.T.T., data curation, formal analysis, investigation, methodology, writing – review and editing, and software; J.D.O., conceptualization, data curation, formal analysis, project administration, visualization, writing – original draft, writing – review and editing, supervision, funding acquisition, and resources.

## Declaration of interests

There are no competing interests between authors.

## STAR★Methods

### Key resources table

**Table:** 

REAGENT or RESOURCE	SOURCE	IDENTIFIER
**Critical commerical kits**		

SepMate™ tubes	StemCell Technologies	Cat No.: SepMate-50(RUO)
Lymphoprep™	StemCell Technologies	Cat No. 18061
EasySep™ CD4^+^CD127loCD25^+^ Isolation Kit	StemCell Technologies	Cat No. 18063
EasySep™ CD4^+^ T Cell Isolation Kit II	StemCell Technologies	Cat No. 17852
EasySep™ Human Monocyte Isolation Kit	StemCell Technologies	Cat No. 19059
ImmunoCult™ DC Differentiation Kit	StemCell Technologies	Cat No. 10985
Genomic DNA Extraction Kit	New England Biolabs	Cat No. T3010L
Lenti-X Provirus Quantitation Kit	Takara	Cat No. 631239
DNA Plasmid Extraction Kit	Qiagen	Cat No. 27104
Cytometric Beads Array Human Cytokine Kit	BD	Cat No. 560484

**Biological samples: Chemicals**		

RPMI 1640	Gibco	Cat No. 11875093
Human AB Serum	Sigma Aldrich	Cat No. 2918
GlutaMAX	Gibco	Cat No. 35050061
Recombinant Interlukin 2 Human	Sigma Aldrich	Cat No. I2644
Immunocult XF T cell Expansion Medium	StemCell Technologies	Cat No. 10891
Expi293 Expression Medium	Gibco	Cat No. A1435101
LV MAX Production Medium	Gibco	Cat No. A3583401

**Experimental models: Cells**		

Expi293F GnTI^−^ cells	Gibco	Cat No. A39240
LV MAX Viral Production cells	Gibco	Cat No. A35347
*E.coli* Top 10	Life Technologies	Cat No. C404003

**Experimental models: Recombinant DNA**		

pHLsec	Addgene	Cat No. 99845
pRSV-Rev	Addgene	Cat No. 12253
pMD2.G	Addgene	Cat No. 12259
pMDL/pRRL	Addgene	Cat No. 12251

**Antibodies**		

Anti-CD4 Pacific Blue	BD	Cat No. 558116
Anti-CD127 PE	BD	Cat No. 561028
Anti-FLAG CF594	Coralite	Cat No. CL594-6608
Anti-CD25 BUV395	BD	Cat No. 564034
Anti-CD69 BV711	BD	Cat No. 563836
Anti-LAP PE-Cy7	Invitrogen	Cat No. 25–9829–42
Anti-GARP BV786	BD	Cat No. 742803
Anti-CD19 APC-Cy7	BD	Cat No. 557791
Cell Trace Violet	Invitrogen	Cat No. C34557
Cell Trace Far Red	Invitrogen	Cat No. C34572
Live Dead NIR	Invitrogen	Cat No. L34975
Live Dead Blue	Invitrogen	Cat No. L23105

**Deposited data**		

Protein Structure	PDB	https://doi.org/10.2210/pdb9yz3/pdb
TCR Data	NCBI	PRJNA1512058

**Software**		

GraphPad Prism 10	GraphPad Prism	Version 10
CCP4	Australian Synchontron	N/A
Coot	Australian Synchontron	N/A
PHENIX	Australian Synchontron	N/A

### Experimental model and study participant details

#### Cells & DNA

All cell lines and DNA used in this study are listed in the key resources table under “Experimental Models: Cells” and “Experimental Models: Recombinant DNA”. The cell lines and recombinant DNA were obtained from external vendors, as indicated in the key resources table. All cell lines were tested by the respective vendors for mycoplasma contamination prior to shipment and were confirmed to be free of mycoplasma contamination.

#### SjD patients and healthy controls

All patients with SjD and healthy controls included in this study were recruited under protocols approved by the Central Adelaide Local Health Network Human Research Ethics Committee (ref. 2008124) and the Monash University Human Research Ethics Committee (ref. 41416). The study was conducted in accordance with the National Statement on Ethical Conduct in Human Research (2007). All participants provided written informed consent prior to participation. Detailed clinical and demographic characteristics of the SjD participants are provided in Table S3. Healthy controls are females aged between 40 and 60 years old and all positive for the HLA-DR3 allele.

### Method details

#### HLA typing and isolation of peripheral blood mononuclear cells (PBMCs) and T Cell subsets

Following informed consent and ethics approval, up to 500 mL of whole blood was collected via venepuncture. An aliquot of 200 μL was used for human leukocyte antigen (HLA) typing, performed by the Australian Red Cross. The remaining volume was processed for peripheral blood mononuclear cell (PBMC) isolation. This study was approved by the Central Adelaide Local Health Network Human Research Ethics Committee (ref. 2008124) and the Monash University Human Research Ethics Committee (ref. 41416).

Blood samples obtained from healthy donors aged 40–60 years were all positive for the HLA-DR3 allele, and from female patients with SjD, aged 49–80 years, who were anti-Ro60 seropositive and carried the HLA-DRB1∗03:01 allele (Table S3). Samples were processed using SepMate™ tubes (StemCell Technologies) for density gradient centrifugation, after which PBMCs were collected for downstream applications. Treg were enriched using the EasySep™ CD4^+^CD127^lo^CD25^+^ Isolation Kit (StemCell, Cat. No: 18063), and conventional CD4^+^ T cells (Tconvs) using the EasySep™ CD4^+^ T Cell Isolation Kit II (Cat. No: 17852). For co-culture assays, monocytes were isolated from PBMCs using the EasySep™ Human Monocyte Isolation Kit and differentiated into mature dendritic cells (DCs) with the ImmunoCult™ DC Differentiation Kit (StemCell Technologies).

#### Identification of the dominant Ro60 T cell epitope and Ro60-reactive TCRs

CD4^+^ T cells were isolated from a healthy donor for heterozygous HLA-DRB1∗03:01 (HLA-DR3) as described above. CD4^+^ T cells were labeled with CellTrace Violet (CTV) and co-cultured with Ro60_225-239_ or Ro60_369-383_ peptide-pulsed DCs at a 1:1 ratio in supplemented RPMI (10% human AB serum, 2% penicillin/streptomycin, 2 mM L-glutamine, 50 μM 2-mercaptoethanol) with 80 U/mL recombinant human IL-2. Co-cultures were maintained at 37 °C, 5% CO_2_ for 6 days. T cell activation and proliferation were assessed by Cytek Aurora using antibodies against CD4, CD25 and CD127. Peptide selection was based on T cells exhibiting the highest proliferative response.

Next, activated T cells were sorted (CD4^+^CTVlow) using a BD Aria cell sorter, adjusted to 1 × 10^6^ cells/mL in 0.04% BSA/PBS, and loaded into the 10X Chromium Controller (10X Genomics) for single-cell RNA sequencing following manufacturer instructions. Gene expression and V(D)J libraries were sequenced on an Illumina NextSeq System and processed with Cell Ranger (10X Genomics), followed by analysis in Loupe Cell Browser (version 4) and Loupe VDJ Browser (version 4). TCR clonotypes with highest expansion were identified for downstream studies.

#### Expression and purification of recombinant HLA-DR3

The cDNAs encoding the extracellular domains of the HLA-DR3 α- and β-chains were separately cloned into the pHLsec expression vector (Addgene plasmid #99845). Both constructs contained C-terminal enterokinase-cleavable fos/jun zipper domains to promote heterodimerization. The β-chain carried a C-terminal His-tag for immobilized metal affinity purification and an N-terminal fusion consisting of a Strep-tag and Factor Xa-cleavable linker to covalently attach target peptides (Ro60, CLIP, CLIP_P6K, or CLIP_P4D). Correct plasmids were sequence verified in E. coli Top10 (Life Technologies) and purified using a DNA Plasmid Extraction Kit (Qiagen).

The recombinant HLA-DR3 with a covalently linked peptide was transiently expressed in Expi293 GnTI^−^ cells (ThermoFisher) using Polyethyleneimine (PEI) (BIO-Scientific) transfection, and soluble protein was harvested from culture supernatant after seven days. The protein was dialyzed into 20 mM Tris, 150 mM NaCl and purified using immobilized metal affinity chromatography (HisTrap HP; Cytiva) followed by size-exclusion chromatography (HiLoad 16/600 Superdex; Cytiva) in 20 mM Tris, 150 mM NaCl. Fractions corresponding to ∼67 kDa were pooled and concentrated for downstream applications.

#### Differential scanning fluorimetry

Thermal stability of recombinant HLA-DR3 protein with mutant CLIP was assessed by differential scanning fluorimetry (DSF) and backreflection using the Prometheus NT.48 by NanoTemper at the Monash Macromolecular Crystallisation Platform (MMCP) in capillaries tubes (10 μL per capillary, run in replicates). Protein samples were prepared in 1× TBS buffer (pH 8) at a final concentration of 0.2 mg/mL. The assay monitored fluorescence emission at 330 nm (buried aromatic residues) and 350 nm (exposed residues), as well as the amount of optical light backreflected through the capillaries from a mirror, as the temperature increased from 20 °C to 95 °C at a ramp rate of 1 °C/min.

#### Peptide loading and purification of HLA-DR3-Ro60 complexes

Recombinant HLA-DR3–CLIP_P4D was digested with Factor Xa (NEB) at a 50:1 protein-to-protease ratio in 20 mM Tris, 150 mM NaCl, 2 mM CaCl_2_ at room temperature for 6 h. The reaction was quenched with 5 mM EDTA and acidified with 50 mM trisodium citrate. Synthetic Ro60_369-383_ peptide (GL Biochem) was added at a 20:1 M ratio and incubated with HLA-DR3 at 37 °C for 72 h to enable peptide exchange.

Peptide-loaded HLA-DR3-Ro60_369-383_ complexes were separated from non-exchanged Strep-tagged HLA-DR3–CLIP_P4D using StrepTactin II beads (IBA) according to the manufacturer’s instructions. Flow-through fractions containing HLA-DR3-Ro60_369-383_ in 100 mM Tris pH 8, 150 mM NaCl, 1 mM EDTA were analyzed by SDS-PAGE to confirm peptide loading and protein integrity.

#### Protein crystallisation and structural determination of HLA-DRB1∗03:01-Ro60_369-383_

For crystallization, HLA-DR3-Ro60_369-383_ was buffer-exchanged into 10 mM Tris pH 7.6, 50 mM NaCl, and 2 mM CaCl_2_, followed by removal of the fos/jun zipper domains with enterokinase (NEB) at room temperature for 16 h. The extracellular HLA-DR3-Ro60_369-383_ was further purified by anion-exchange chromatography (HiTrap Q HP; Cytiva) and concentrated to 4 mg/mL for crystallization trials at the Monash Macromolecular Crystallisation Platform.

Crystals of HLA-DR3-Ro60_369-383_ were obtained in 0.2M of sodium malon salt and 20%w/v polyethylene glycol (PEG) 3350. Crystals were soaked briefly in 20% glycerol as cryoprotectant before flash-cooling in liquid nitrogen. Diffraction data were collected on the MX2 beamline at the Australian Synchrotron. Data were processed with XDS and scaled and merged using Aimless. Molecular replacement was performed in PhaserMR (CCP4) using the coordinates of PDB entry 1A6A as the search model. Model building and refinement were carried out in Coot and PHENIX, with final validation in MolProbity. Crystallographic data have been deposited in the Worldwide Protein DataBank, and a summary of data collection and refinement statistics is provided in Table S2.

#### Production of lentivirus containing genes encoding Ro60-reactive TCR

The 3rd generation lentiviral plasmids including pMD2.G, pRSV.rev, pMDL/pRRL (Addgene), and FLAG (D DYKDDDDK) tagged **Ro60-reactive** TCR encoding transfer plasmids were transfected into LV MAX™ cells (ThermoFisher) at a molar ratio of 1:2:1:4 using the LVMAX™ Transfection Kit (ThermoFisher). At 72 h post-transfection, the transfected cells were pelleted by centrifugation at 1300xg for 10 min and filtered using 0.45 μm low-binding protein PVDF vacuum-driven filter (Merck). The filtered culture supernatant, containing lentiviral particles, was collected and subjected to ultracentrifugation at 19,000 rpm (Hitachi, P28S Rotor) for 2 h at 4°C, using a 20% sucrose buffer composed of 20% w/v sucrose, 100 mM NaCl, 1 mM EDTA, and 20 mM HEPES. The resulting viral pellet was resuspended in Hanks' Balanced Salt Solution (Sigma Aldrich) and stored at −80°C. Functional titration of lentiviral vectors measured in TU/mL was performed on J76 Jurkat cells.

#### Lentiviral transduction of T cells

Isolated Treg and Tconv cells were transduced with the lentivirus at a density of 50,000 to 100,000 cells per well together with lentiboost (Sirion, Cat No: SB-A-LF-901–01) and protamine sulfate (Sigma-Aldrich, Cat No: P4505-5G) added at concentrations of 1:100 and 8 μg/mL, respectively for effective transduction. Lentivirus was added at various multiplicity of infection (MOI), and the plate was subjected to spinoculation at 1500 × g for 2 h at 31°C. Afterward, the plate was incubated at 37°C with 5% CO_2_ and 95% humidity for 5 days. Transduction efficiency was assessed by measuring green fluorescent protein (GFP) expression, and TCR surface expression was evaluated using an anti-FLAG antibody targeting the FLAG-tagged Ro60-TCR, both analyzed on a Cytek Aurora flow cytometer.

#### qPCR determination of vector copy number (VCN)

Transduced Treg cells were harvested, and genomic DNA (gDNA) was extracted using the Monarch DNA Extraction Kit (New England Biolabs, Cat No: T3010L). Ro60-TCR vector copy number was determined by qPCR (QuantStudio^TM^ 7 Flex, ThermoFisher) using the Lenti-X Provirus Quantitation Kit (Takara, Cat No: 631239) according to the manufacturer’s protocol.

#### Suppression and proliferation assays

Suppression and proliferation assays were performed as described by Eggenhuizen et al.,^30^ using samples from patients with SjD only. For the suppression assay, Cell Trace Far Red (CTFR)-labelled Treg were co-cultured at varying ratios with Cell Trace Violet (CTV)-labelled Tconv, PBMCs, or CD25^−^ T cells, along with Ro60_369–383_ peptide-pulsed HLA-DR3^+^ B-lymphoblastoid cell lines (BLCLs). The culture medium consisted of RPMI 1640 (ThermoFisher, Cat No: 11875093) supplemented with 10% human AB serum, 50 μM 2-mercaptoethanol, 100 U/mL penicillin-streptomycin, 1× GlutaMAX, and 10 mM HEPES. BLCLs were pulsed with Ro60_369–383_ peptide at 37°C for 2 h. Labeled Tconv/PBMCs/CD25^-^ and Treg were assessed for proliferation. Co-cultures were incubated at 37°C with 5% CO_2_ for 120 h. The proliferation assay followed the same setup, excluding Tconv/PBMCs/CD25^-^ cells. Flow cytometry (Cytek Aurora) was used to assess cell proliferation in both assays, with polyclonal (mock) Treg as controls.

#### Cytokine assay

Supernatant from the both assays mentioned above were ran for detection of IL-10 and IFN-γ. This experiment was executed using Becton Dickinson (BD)^TM^ Cytometric Beads Array Human Th1/Th2/Th17 Cytokine Kit (BD, Cat No.: 560484) and analyzed via BD LSRFortessa^TM^ Cell Analyzer.

### Quantification and statistical analysis

Statistical analyses and data visualization were performed using GraphPad Prism 10 (GraphPad Software). For comparisons between two groups, two-tailed paired or unpaired t-tests were used as appropriate. Paired t-tests were used for donor-matched experiments in which the same donor was assessed under different experimental conditions, whereas unpaired t-tests were used for comparisons between independent groups. For comparisons involving three or more groups, one-way ANOVA was performed. Statistical significance was defined as *p* < 0.05. Significance levels are indicated as follows: ∗*p* < 0.05, ∗∗*p* < 0.01, ∗∗∗*p* < 0.001, and ∗∗∗∗*p* < 0.0001. The statistical test used, number of biological replicates and definition of error bars are indicated in the corresponding figure legends.
