# Supplementary material for: Tregs transduced with a Ro60-reactive TCR have increased immunosuppressive functions in Sjögren’s disease
Source: iScience. 2026 Sep 28;29(10):117642. doi: 10.1016/j.isci.2026.117642 (PMC13651826; doi:10.1016/j.isci.2026.117642)
Supplement: Supplementary file 1 [file mmc1.pdf]

iScience, Volume *29*

Supplemental information

Tregs transduced with a Ro60-reactive TCR have increased immunosuppressive functions in Sjögren's disease

Zhi Feng Sherman Lim, Rachel M.Y. Cheong, Fabien B. Vincent, Janet Chang, Khai L. Loh, Hanim Halim, Geoffrey Kwai-Wai Kong, Elean S.V. Tay, Cheng Rui Peh, Xiaoyu Jia, Maureen Rischmueller, Yi Tian Ting, and Joshua D. Ooi

Document S1. Figures S1–S9 and Tables S1–S3.

Figure S1, related to Figures 1–3 and the STAR Methods, presents protein expression data for the various recombinant proteins evaluated in this study. **Figure S2**, related to Figure 5 and the STAR Methods, shows representative flow cytometry plots for the different multiplicities of infection (MOIs) tested. **Figures S3–S5**, related to Figure 6 and the STAR Methods, summarize transduction efficiency and surface expression data for all healthy controls and patients with Sjögren's disease (SjD). **Figures S6–S9**, related to Figure 7 and the STAR Methods, present proliferation and suppression assay results from all SjD patients and healthy controls included in the study.

Table S1, related to Figure 4 and the STAR Methods, provides the sequence information for TCR1. **Table S2**, related to Figure 3 and the STAR Methods, contains the processed data collected from the Australian Synchrotron. **Table S3**, related to Figures 6 and 7, summarizes patient demographic information and serological test results.

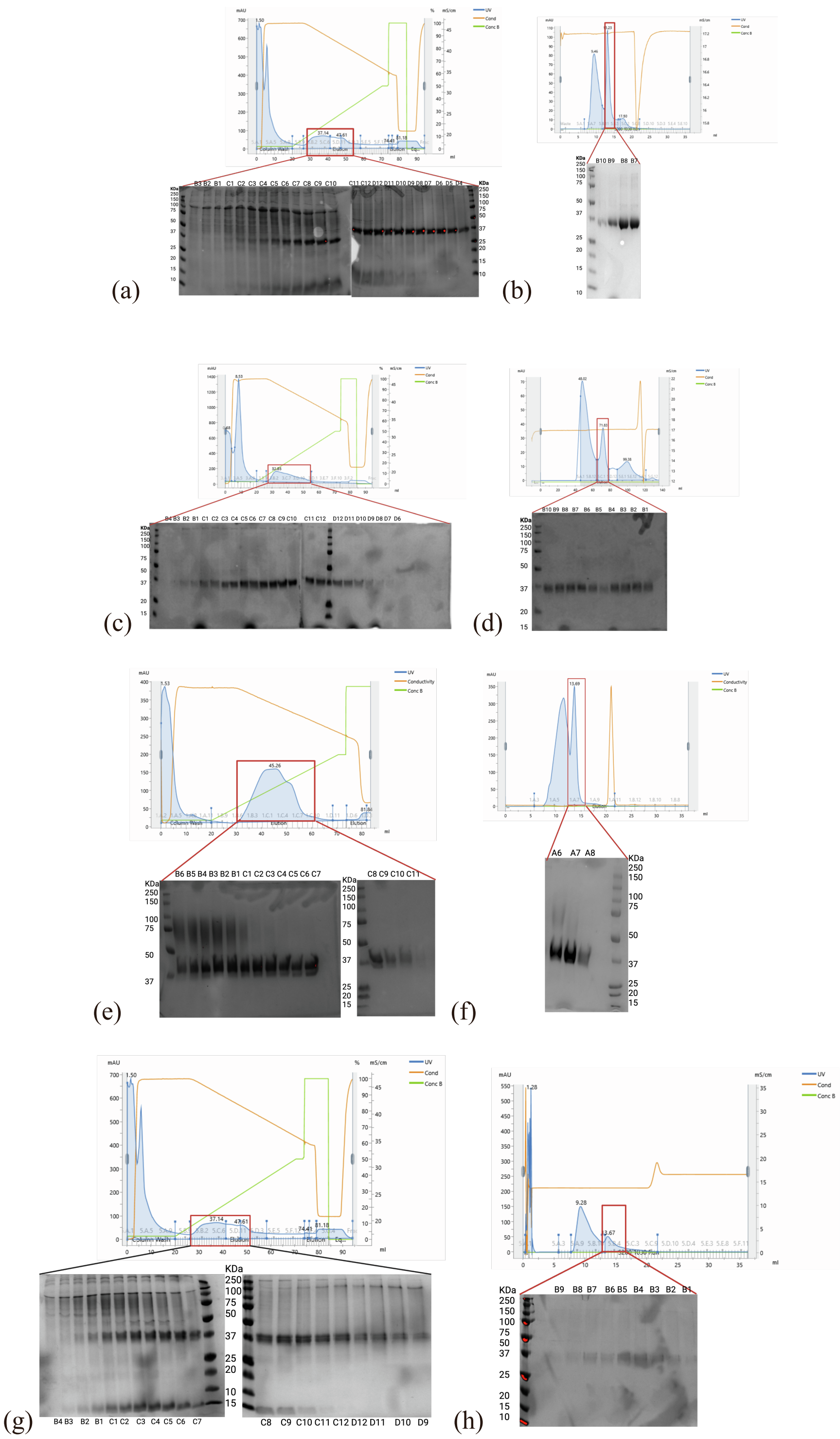

Figure S1. Purification profile of various HLADRB01*03:01 proteins

(a, c, e, and g) Nickel affinity chromatography purification profiles of HLA-DRB103:01–CLIP, HLA-DRB1*03:01–CLIP–P6K, HLA-DRB1*03:01–CLIP–P4D, and HLA-DRB1*03:01–Ro60$_{369–383}$, respectively. (b, d, f, and h) The corresponding size-exclusion chromatography (SEC) profiles are shown. Two protein bands were observed at approximately 35 and 37 kDa, corresponding to the β and α chains of HLA-DRB1*03:01, respectively. Each binary complex contains a hexahistidine tag, enabling initial purification by nickel affinity chromatography to remove non-tagged contaminants. The eluted fractions were subsequently subjected to SEC to further separate the target complexes from protein aggregates.

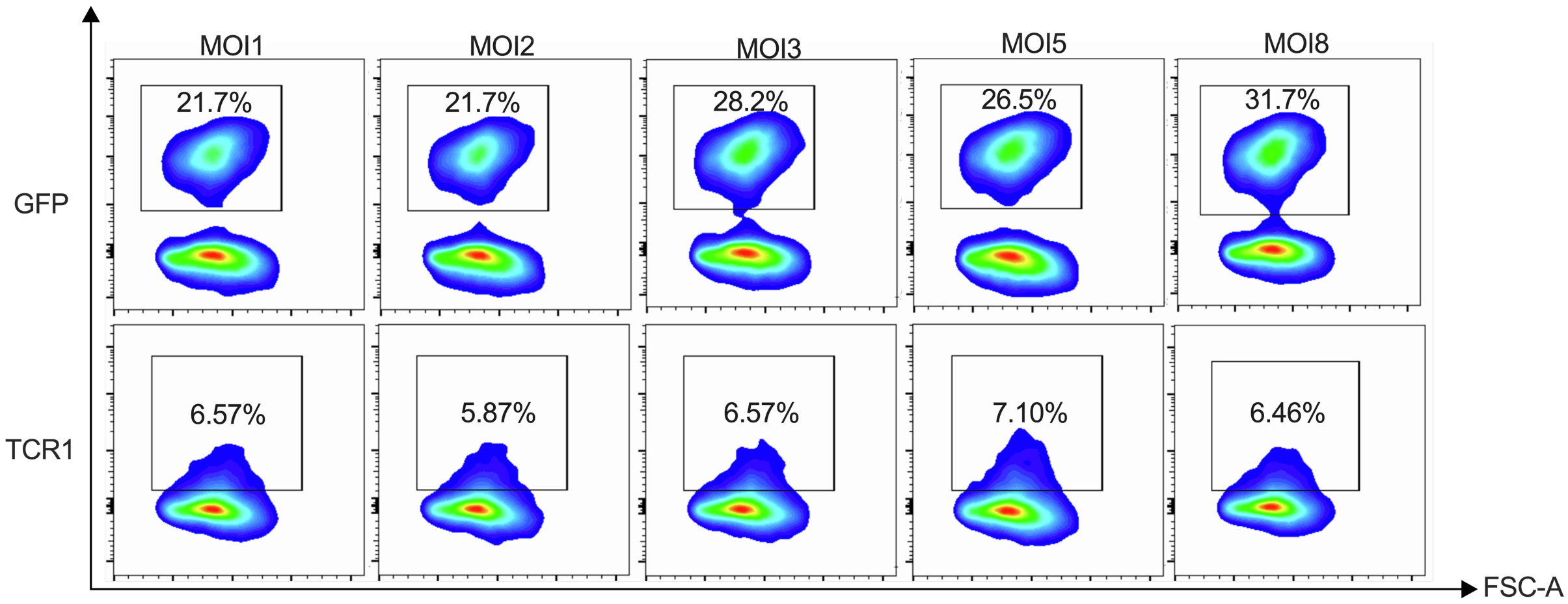

Figure S2. Viral MOI test at various MOI 1, 2, 3, 5, and 8.
This figure illustrates the surface expression and transduction efficiency of various MOIs, using 1×10^5 cells per condition. The results indicate that increasing transduction efficiency does not correlate with higher surface expression of TCR1, as surface expression levels remained consistent across all MOIs tested.

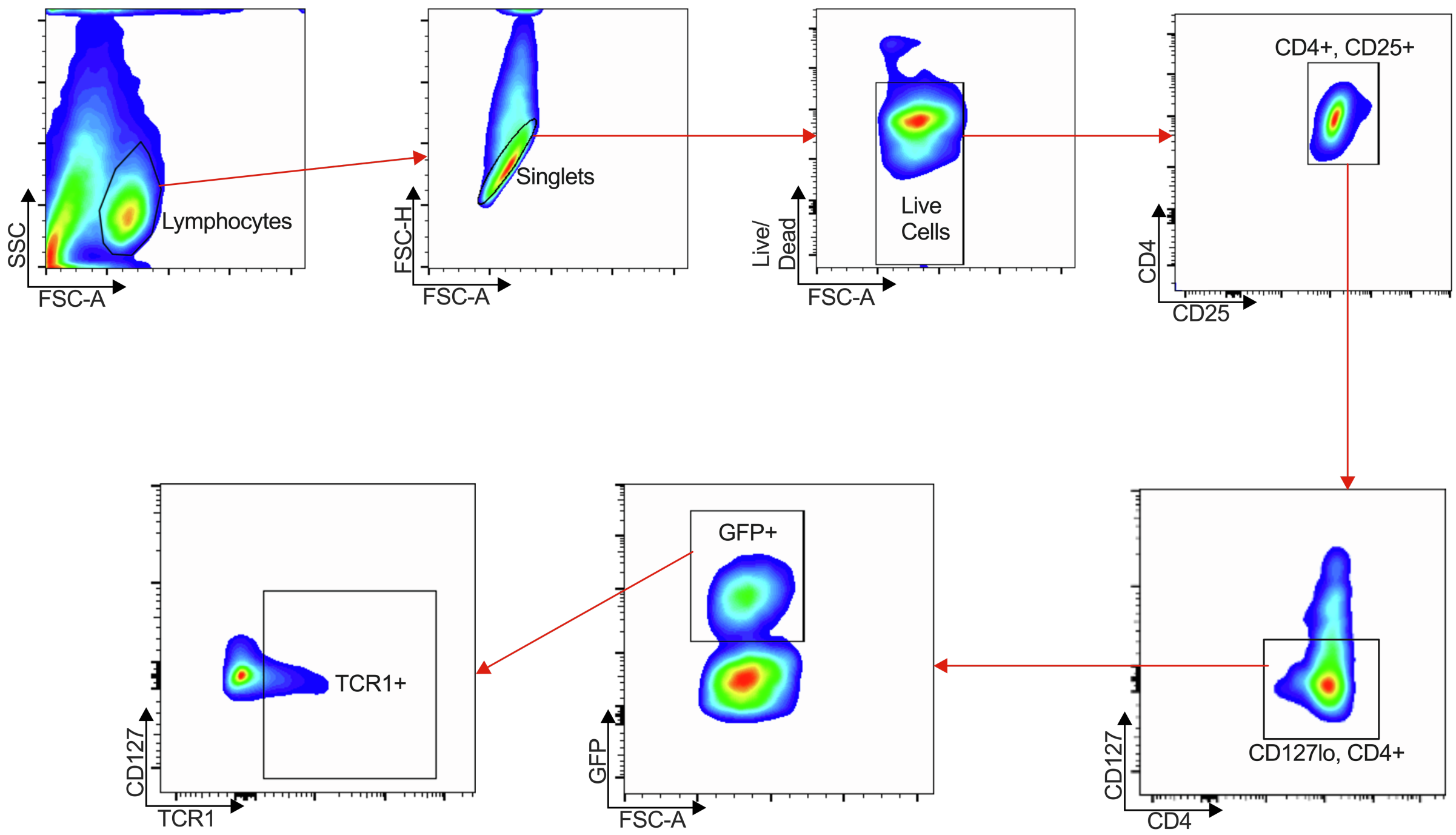

Figure S3. Gating Strategy for transduction efficiency and surface expression.
Gating strategy was employed to assess transduction efficiency and surface expression. Lymphocytes were first gated based on forward scatter (FSC) and side scatter (SSC), followed by gating for singlets and live cells using Near Infrared (NIR) staining. Among the live cells, Treg cells were identified from the CD4hi and CD25hi population. Within this subset, CD127lo cells were further gated. From this population, GFP-positive cells were selected, and the surface expression of TCR1 was evaluated within the GFP-positive cell population.

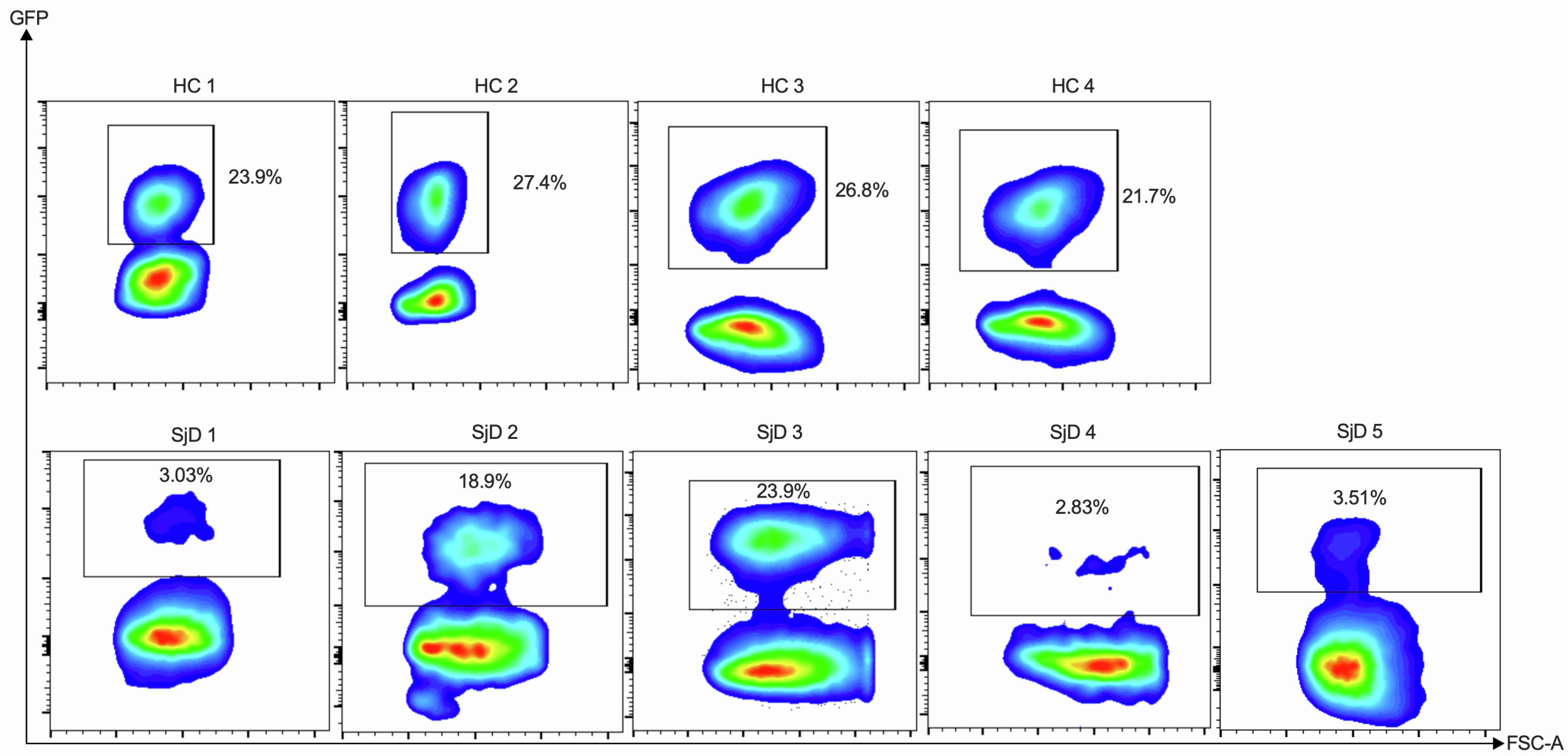

Figure S4. TCR1 is equally transducible in all healthy controls and SjD patients.
Flow cytometry plots depicting the transduction efficiency of TCR1 in healthy control individuals and patients with SjD who carry the HLA-DRB1*03:01 allele. The plots offer a detailed comparison of transduction efficiency levels between the two groups, highlighting any differences in the efficiency of TCR1 transduction. These data provide a visual representation of the transduction process and enable a direct comparison of its effectiveness in individuals with SjD versus healthy controls.

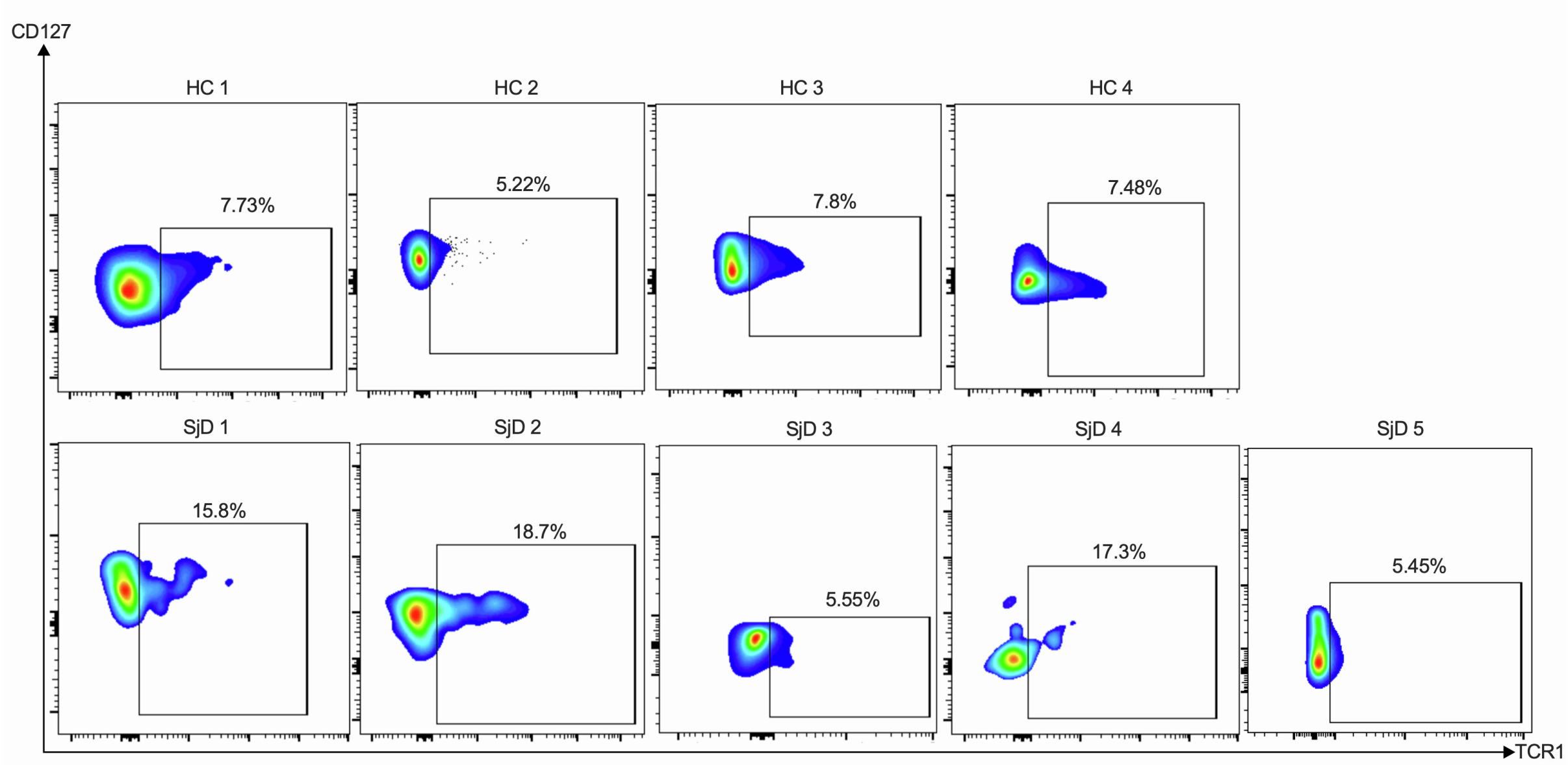

Figure S5. TCR1 is equally surface expressible in all healthy controls and SjD patients.
Flow cytometry plots illustrating the surface expression of TCR1 in healthy control individuals and patients with SjD who possess the HLA-DRB01*03:01 allele. These plots offer a detailed comparative analysis of TCR1 expression levels between the two groups, providing a clear visualization of any differences in surface expression. The data enable an assessment of the relative abundance of TCR1 on the cell surface in both healthy controls and SjD patients.

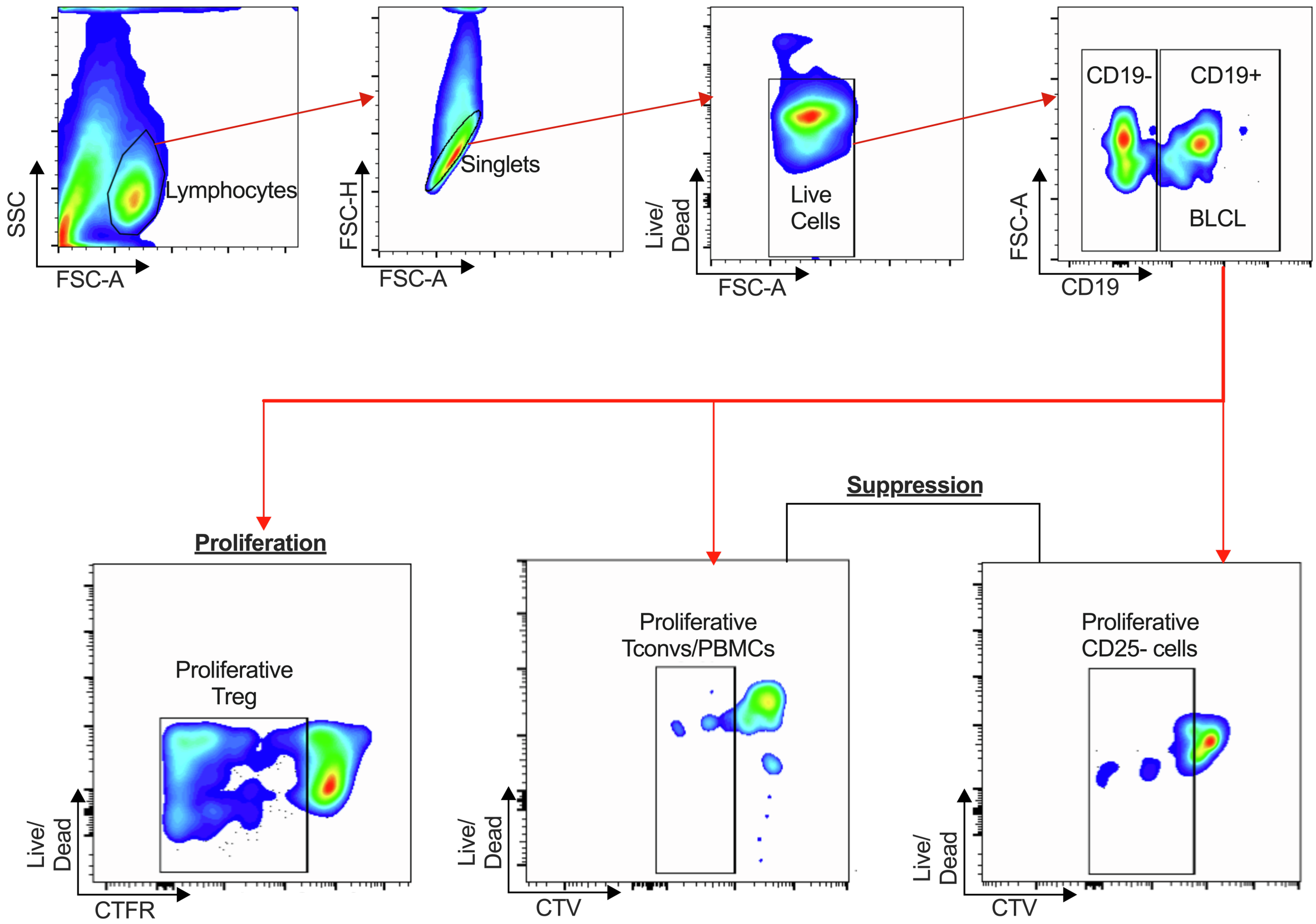

Figure S6. Gating Strategy for suppression and proliferation assay.
Gating strategy was implemented to assess the proliferation of Treg cells and the suppression of Tconv cells, PBMCs, or CD25-negative cells. Singlet live lymphocytes were gated as described in Supplementary Figure 2. Within the live cells, CD19-positive and CD19-negative populations were identified, with the CD19-negative subset representing non-BLCL cells. From the CD19-negative population, Cell Trace Violet (CTV) staining was used to assess the proliferation of Tconv cells, PBMCs, or CD25-negative cells, while Cell Trace Far Red (CTFR) staining was used to evaluate the proliferation of Treg cells.

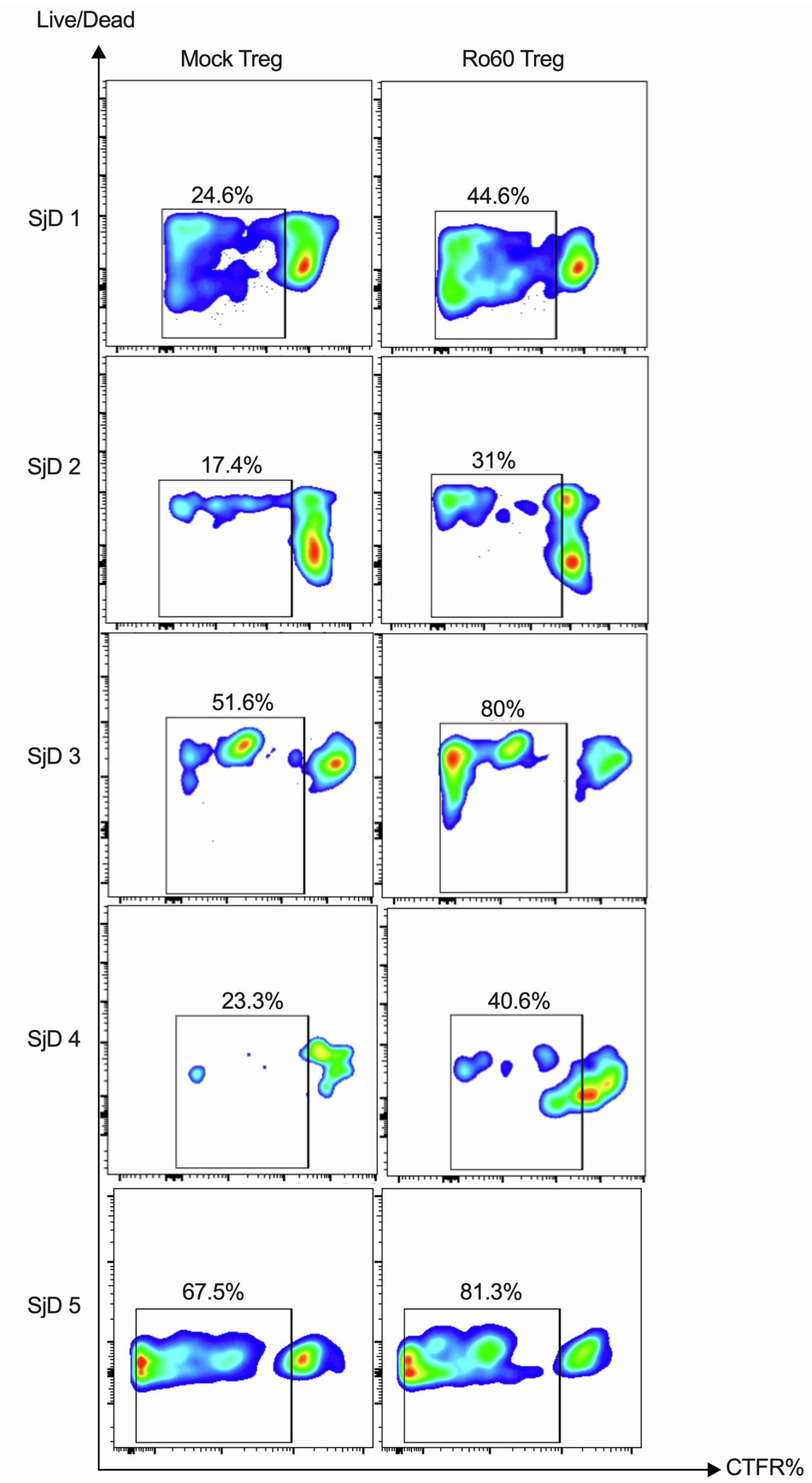

(a)

Patient	Mock Treg (Proliferative Treg %)	Ro60-Treg (Proliferative Treg %)
SjD 1	24.6	44.6
SjD 2	17.4	31
SjD 3	51.6	80
SjD 4	23.3	40.6
SjD 5	67.5	81.3

(b)

Figure S7. Engineered Treg with TCR1 are more proliferative than Treg without TCR1
(a) The proliferation assay, as shown in the figure above, was conducted to evaluate the activation and proliferative responses of Ro60-Treg in comparison to Treg that do not express the TCR1 (Mock Treg), using Cell Trace Far Red (CTFR) as a marker. This assay was performed across all patients diagnosed with SjD to assess how well the engineered Tregs, with the TCR1 modification, were activated in response to $Ro60_{369\text{-}383}$. The *in vitro* assay was set up using a co-culture system, where the Treg were cultured alongside with BLCL, and proliferation was measured after a 7-day incubation period. The CFTR dye was utilized to track the proliferation of Treg, with dilution of the dye indicating cell division. The results from this assay provide important information on the activation and expansion of engineered Treg relative to the standard Treg that lack the TCR1. (b) A table summary is included in this supplementary figure.

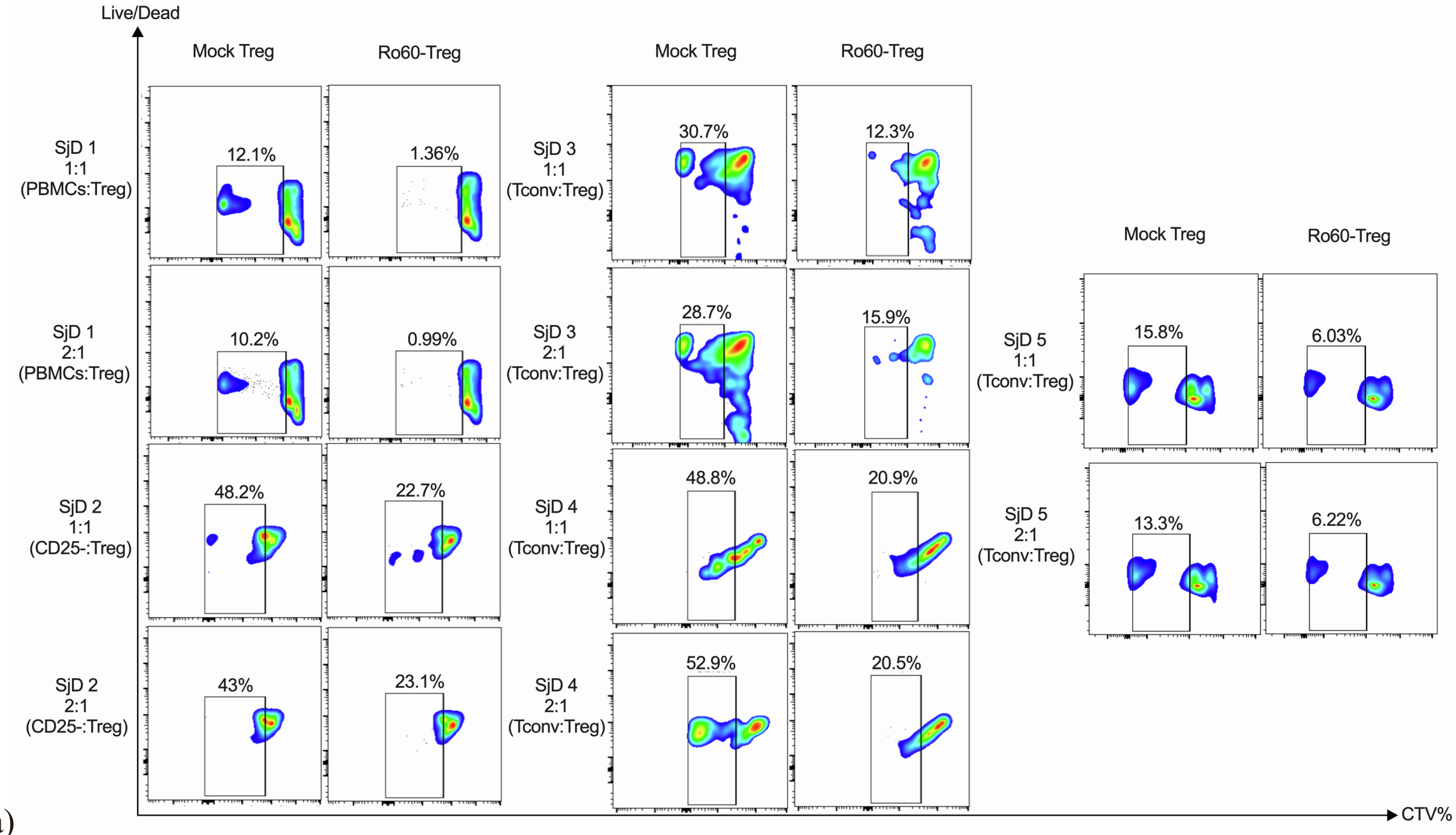

(a)

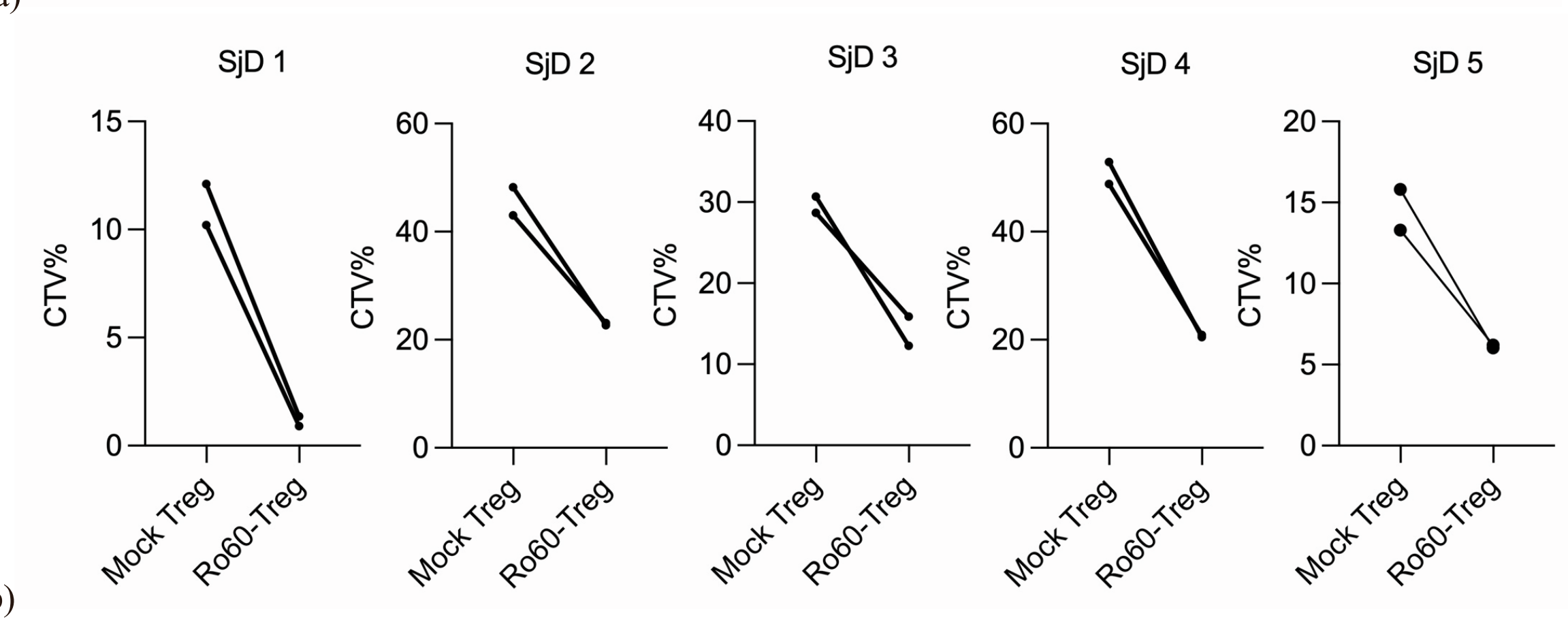

(b)

(c)

Patient	Ratio (Tconv : Treg)	Mock Treg (Proliferative Tconv %)	Ro60-Treg (Proliferative Tconv %)
SjD 1	1:1	12.1	1.36
	2:1	10.1	0.99
SjD 2	1:1	48.2	22.7
	2:1	43	23.1
SjD 3	1:1	30.7	12.3
	2:1	28.7	15.9
SjD 4	1:1	48.8	20.9
	2:1	52.9	20.5
SjD 5	1:1	15.8	6.03
	2:1	13.3	6.22

(c)

Figure S8. Engineered Treg with TCR1 shown better suppressive capacity than those without TCR1 in SjD patients

(a) Flow cytometry plots illustrating the suppression assays conducted on five patients with SjD. These assays were performed at two different Tconv/PBMCs/CD25- to Treg ratios: 1:1 and 2:1. The plots demonstrate the detailed assessment of immune cell interactions and regulatory T cell activity in response to varying T cell ratios. (b) Each individual SjD patients, comparing suppressive capacity of Ro60-Treg and Mock Treg are depicted in the graph showing decreased trend of CTV%. (c) A table summary is included in this supplementary figure.

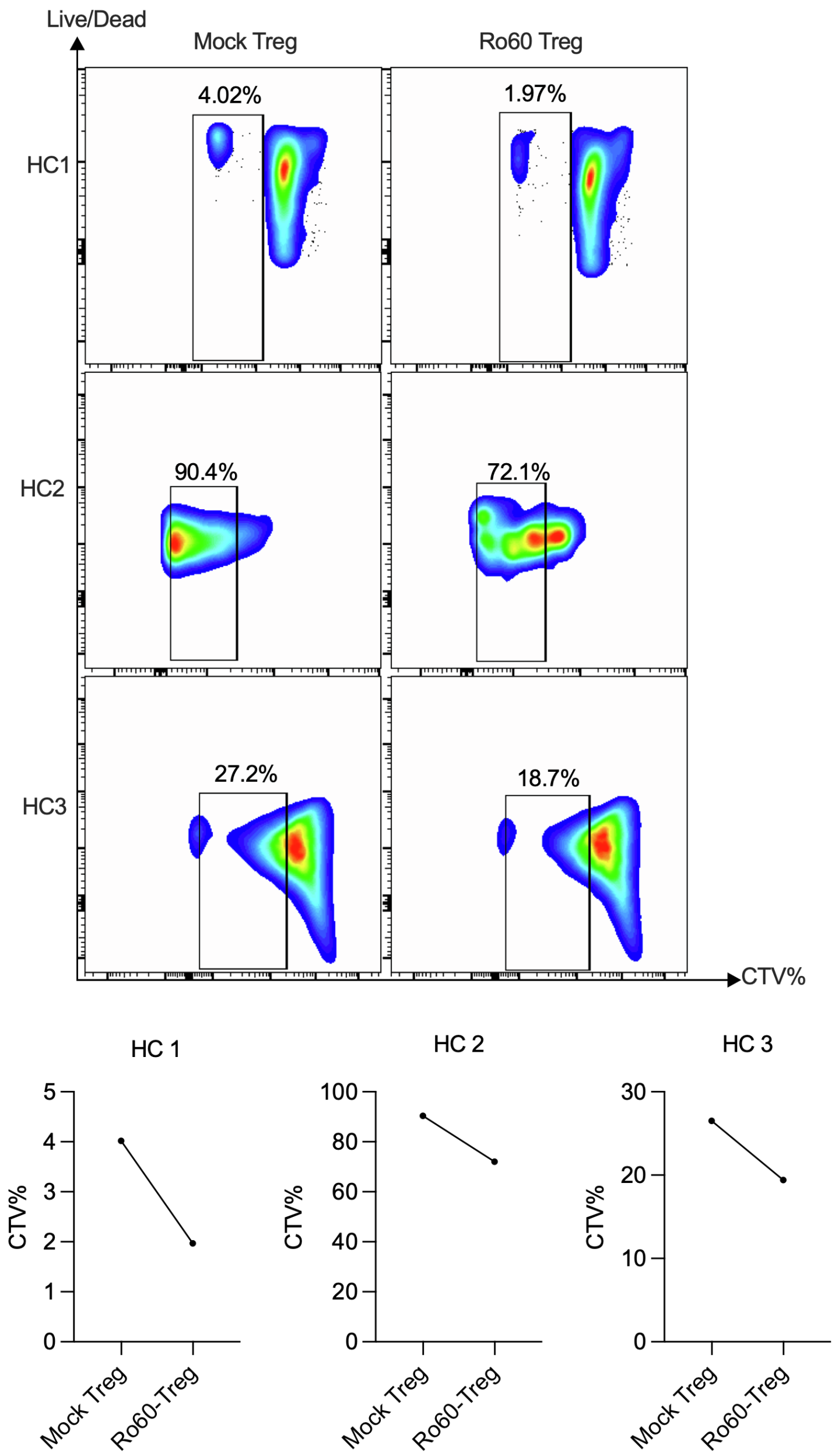

Figure S9. **Engineered Treg with TCR1 shown better suppressive capacity than those without TCR1 in healthy control (HC)**

Representative flow cytometry plots depicting Tconv cell proliferation following co-culture with Treg in response to $Ro60_{369\text{-}383}$ peptide in three HCs. Tconv cells were co-cultured with either Ro60-Treg cells expressing TCR1 or Mock Treg cells lacking TCR1 at a 1:1 Tconv ratio. Tconv cell proliferation was assessed by CTV dilution, with decreasing CTVlo (CTV%) fluorescence indicating increased proliferation.

Table S1. Variable gene and CDR3 sequence of TCR1

TCR	Variable Gene	CDR3
TCR1	TRAV12-1<br>TRBV27	CVVPPGGGFKTIF<br>CASRTRTASYGYTF

Table S2. Crystallographic data collection and refinement statistics.

Data Collection	
Space Group	C121
Unit Cell a,b,c (Å)	110.62 96.84 102.60
Unit Cell α,β,γ (°)	90.01 98.60 89.91
Resolution Range	40.32 - 2.87
Total reflections	130606 (3808 – 17309)
Unique reflections	30377 (866 – 4048)
R_{merge}	0.136 (0.054 – 0.776)
Rpim	0.112 (0.044 – 0.645)
Completeness	99.5% (98.8 – 99.9)
Mean I/SigmaI	8.1 (1.8)
$Cc_{1/2}$	0.989 (0.992 – 0.598)
Multiplicity	4.3 (4.4 – 4.3)
Mean $\boldsymbol{\sigma}$(I/I sd)	8.1 (21.2 – 1.8)
R-work	0.2370 (0.2376)
R-free	0.2989 (0.3011)
RMS (Bonds)	0.0051
RMS (Angles)	1.4602
Ramanchandran favored (%)	91.59
Ramanchandran outliers (%)	0.81

Table S3. Five SjD patients details on birth year, gender, ESSDAI, serological test, extra-glandular manifestation and current treatment received.

**SjD Patients**	SjD 1	SjD 2	SjD 3	SjD 4	SjD 5
**Year**	1957	1946	1976	1950	1948
**Gender**	Female	Female	Female	Female	Female
**Antibodies**	Ro60+, Ro52+, La+	Ro60+, Ro52+, La-	Ro60+, Ro52+, La+	Ro60+, Ro52+, La-	Ro60+ Ro52+ La+
**ESSDAI**	6	5	3	0	6
**Extra-glandular Manifestation**	Tubulointerstitial nephritis, Renal tubular acidosis, Nephrocalcirosis and Vasculitis	Inflammatory arthritis	NIL	Arthralgia	Myalgia, Vasculitic skin rash
**Treatment**	NIL	Hydroxychloroquine	Hydroxychloroquine	NIL	NIL

EULAR Sjögren's syndrome disease activity index, ESSDAI
